# Natural Design of a Stabilized Cross‐β Fold: Structure of the FuA *FapC* from *Pseudomonas* Sp. UK4 Reveals a Critical Role for Stacking of Imperfect Repeats

**DOI:** 10.1002/adma.202505503

**Published:** 2025-06-11

**Authors:** Yanting Jiang, Samuel Peña‐Díaz, Zhefei Zhang, Anders Ogechi Hostrup Daugberg, Marcos López Hernández, Janni Nielsen, Qiaojie Huang, Shenghan Qin, Morten K. D. Dueholm, Mingdong Dong, Jan Skov Pedersen, Qin Cao, Daniel E. Otzen, Huabing Wang

**Affiliations:** ^1^ Department of Clinical Laboratory the First Affiliated Hospital of Guangxi Medical University Key Laboratory of Clinical Laboratory Medicine of Guangxi Department of Education Guangxi Key Laboratory of Enhanced Recovery after Surgery for Gastrointestinal Cancer Shuangyong Road 6, Guangxi Zhuang Autonomous Region Nanning 530021 China; ^2^ Interdisciplinary Nanoscience Center (iNANO) Aarhus University Gustav Wieds Vej 14 Aarhus C 8000 Denmark; ^3^ Department of Chemistry and Bioscience Aalborg University Fredrik Bajers Vej 7H Aalborg OE 9220 Denmark; ^4^ Department of Chemistry Aarhus University Langelandsgade 140 Aarhus C 8000 Denmark; ^5^ Bio‐X Institutes Key Laboratory for the Genetics of Developmental and Neuropsychiatric Disorders Ministry of Education Shanghai Jiao Tong University Shanghai 200030 China; ^6^ Department of Molecular Biology and Genetics Aarhus University Universitetsparken 2 Aarhus C 8000 Denmark

**Keywords:** cross‐seeding, FuA, Greek key motif, sequence motifs, site‐directed mutagenesis, stable protein self‐assembly

## Abstract

An essential structural component of bacterial biofilms is functional amyloid (FuA), which also has great potential as an engineerable nano‐biomaterial. However, experimentally based high resolution structures of FuA that resolve individual residues are lacking. A fully experimentally based 3.2 Å resolution cryo‐electron microscopy density map of the FuA protein *FapC* from *Pseudomonas* sp. UK4 is presented, which reveals a Greek key‐shaped protofilament. The structure supports bioinformatic identification of conserved motifs and is broadly consistent with the AlphaFold prediction but with important modifications. Each *FapC* monomer consists of three imperfect repeats (IRs), with each repeat forming one cross‐β layer. An array of highly conserved Asn and Gln residues with an extensive H‐bonding network underpins this conserved Greek key‐shape and reveals the role of heterogeneous cross‐β stacking in amyloid cross‐seeding. The covariation of residues in the hydrophobic core among different IRs suggests a cooperative monomer folding process during fibril elongation, while heterogeneous stacking of IRs reduces charge repulsion between layers to stabilize the monomer fold. The *FapC* fibrils show intrinsic catalytic activity and strain‐dependent nanomechanical properties. Combined with mutagenesis data, the structure provides mechanistic insights into formation of *FapC* FuA from disordered monomers and a structural foundation for the design of novel biomaterials.

## Graphical abstract

We report a 3.2 Å cryoEM structure of functional amyloid protein FapC from Pseudomonas sp. UK4, an essential component of bacterial biofilm, which reveals a Greek key‐shaped protofilament, supports bioinformatically determined motifs, nuances AlphaFold predictions, emphasizes heterogeneous cross‐β stacking in amyloid cross‐seeding and shows strain‐dependent nanomechanical properties. FapC fibrils are intrinsically catalytic. This provides a structural foundation to design novel biomaterials.

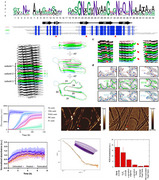

## 1 Introduction

Proteins self‐assemble in many different ways. A striking example is the amyloid fold, which stacks multiple copies of a given protein molecule on top of each other, stabilized by intermolecular β‐sheet bonds at the backbone level and steric zippers interdigitating at the side chain level.^[^ ^1^, ^2^ ^]^ These repetitive and highly complementary contacts assure high stability for the amyloid state, in some cases even exceeding that of globular proteins when a significant part of the protein is integrated into the core of the amyloid structure.^[^ ^3^ ^]^ Though amyloid is often associated with neuronal cell death in neurodegenerative diseases such as Alzheimer's and Parkinson's,^[^ ^4^ ^]^ numerous proteins form so‐called functional amyloid (FuA). Functions range from signal transduction, information storage and transmission, storage and controlled release of peptide hormones to strengthening of bacterial biofilm.^[^ ^5^ ^]^ The highly repetitive and stable amyloid surface also lends itself to diverse kinds of functionalization which has been used to generate novel and signal‐responsive biomaterials.^[^ ^6^ ^]^ We have suggested that they could be engineered for catalytic activity embedded within the actual amyloid structure,^[^ ^7^ ^]^ building on the intrinsic (though low) hydrolytic activities reported for a range of different amyloids.^[^ ^8^, ^9^, ^10^, ^11^ ^]^ Unsurprising in view of this biological usefulness, FuA distinguishes itself by being optimized for self‐assembly. This contrasts with pathological amyloid, where self‐assembly is an unfortunate and unwanted corollary of the aggregating protein's physical–chemical properties and therefore can proceed along multiple different pathways to different amyloid structures, a phenomenon known as polymorphism.^[^ ^12^ ^]^ Pathological amyloid fibril structures differ for the same protein sequence prepared in different conditions or even under similar conditions,^[^ ^13^ ^]^ both within and between protofibrils^[^ ^14^, ^15^ ^]^ and are also sensitive to mutagenesis.^[^ ^16^ ^]^

The biological and physical principles of what might be termed good amyloid production (GAP) are particularly well understood in biofilm‐forming bacteria, which use FuA as structural components in their extracellular polymeric substance matrix to provide rigidity and shape to the biofilm.^[^ ^17^, ^18^ ^]^ FuA is found in a wide range of bacteria.^[^ ^19^, ^20^, ^21^ ^]^ The two most well studied examples are the curli system in *Escherichia coli* and the *fap* system in *Pseudomonas*, in which the major aggregating protein is *CsgA* ^[^ ^22^ ^]^ and *FapC*,^[^ ^23^ ^]^ respectively. In both cases, the same amyloid system is also found in a large number of related species, providing useful evolutionary insights into the development and optimization of these systems.^[^ ^24^, ^25^, ^26^ ^]^ GAP is assured in two ways: first, a carefully controlled system for the export and spatially defined aggregation of the major amyloid protein on the bacterial cell surface and second, a simple but effective organization of the amyloid protein sequence. As regards biogenesis, both curli and *fap* consist of operons which include a dedicated outer membrane export protein (*CsgG* ^[^ ^27^ ^]^ or *FapF* ^[^ ^28^ ^]^) along with associated stabilizing proteins, a periplasmic chaperone to maintain *CsgA*/*FapC* in the unfolded and unaggregated state and a nucleator protein on the bacterial outer membrane which promotes efficient amyloid formation by the amyloidogenic protein once secreted.^[^ ^29^ ^]^ As regards sequence, both *CsgA* and *FapC* consist of several imperfect repeats (IRs) of length ≈20 and ≈35–40 residues, respectively. Each of these repeats has been predicted to form a β‐hairpin which is stabilized internally by interdigitating side chains from each strand and between repeats by backbone hydrogen bonds;^[^ ^17^, ^30^ ^]^ consequently, the repeats can form easily by local interactions and can be stacked indefinitely to form amyloid, leading to a robust and well‐controlled assembly process driven by primary nucleation followed by elongation. This again contrasts with pathological amyloid, whose self‐assembly tends to involve secondary processes such as fragmentation and secondary nucleation.^[^ ^31^, ^32^ ^]^ Fragmentation can however be enhanced in *FapC* by removal of one or more IRs.^[^ ^33^ ^]^

*CsgA* can be considered the minimal amyloid structural unit, since essentially the whole of the sequence is integrated into a simple and cylindrical amyloid structure in the form of a β‐solenoid. By contrast, *FapC*’s longer repeats hint at a more complex structure and each imperfect repeat is linked by linkers of highly variable lengths, ranging from ≈30 to ≈260. In the case of *CsgA*, the number of repeats varies greatly but nevertheless reveals an underlying conserved motif, in which hydrophobic residues point inward to form the core between strands in each repeat, while the externally oriented side chains on the amyloid surface are more varied in composition, though with a greater tendency to be hydrophilic.^[^ ^16^ ^]^ The same study solved the structure of *CsgA* by a combination of cryo‐electron microscopy (cryo‐EM) and computational simulations. The predicted model for a *CsgA* variant with 15.5 repeats fitted the cryo‐EM map with a resolution of 7.6 Å, and this was later improved to 4–6 Å in *E. coli CsgA* with 5 repeats.^[^ ^34^ ^]^ The structures revealed canonical β‐solenoid fold with a conserved cross‐β amyloid kernel, each imperfect repeat forms a layer of multiple β‐strands. The imperfect repeat layers stack on top of each other through parallel hydrogen bonding between β‐strands within β‐sheet, while the interface between the strands within the layer leads to a compact hydrophobic core. However, the low resolution means that the cryo‐EM map does not provide side chain details and interface details between monomers and does not distinguish individual IRs.

In contrast to *CsgA*, the structure of *FapC* has not yet been solved, though AlphaFold (AF) predictions suggest the presence of a Greek key motif.^[^ ^17^, ^35^ ^]^ Here, we present the structure of *FapC* solved entirely by cryo‐EM at 3.2 Å. This level of resolution allows us to identify individual residues and thus distinguish the different IRs unambiguously in the structure. As we have summarized in a recent review,^[^ ^36^ ^]^ the structure of *FapC* from *Pseudomonas* sp. UK4 forms a Greek key‐shaped protofilament of cross‐β structure as predicted by AF3. However, the experimental data show that the β‐strands are tilted out‐of‐plane, while AF3 predicts them to be in‐plane. The staggered β‐strands favor 3D interactions through hydrophobic interactions and hydrogen bonds. Importantly, the three IR layers can be distinguished, thanks to the resolution of the density map of individual side chains. The *FapC* structure is fully consistent with the highly conserved sequence motif that we here identify for the *FapC* IRs. Mutational analysis of highly conserved Asn and Gln residues confirm the extensive H‐bonding network which underpins the stability of this conserved Greek key‐shape. Charge mutations indicate that the heterogeneous stacking of different IRs within the same monomer unit may also reduce charge repulsion between layers to stabilize the monomer fold. Strikingly, destabilization of the amyloid structure by mutation of highly conserved residues involved in an extensive hydrogen‐bonding network increases the protein's tendency to fragment during self‐assembly, consistent with the effects of the removal of entire IRs, although the end‐point fibrils are just as stable as wild‐type (wt) *FapC* UK4. We compare the structural and functional properties of *FapC* UK4 with those of three other related *Pseudomonas* strains and find that *FapC* UK4 represents a minimalistic amyloid fold entirely made up of a robust amyloid core. The other strains have more extensive linker sequences which makes them mechanically softer and increase their width, although their underlying stabilities are essentially identical. Finally, we show that the *FapC* amyloid is intrinsically catalytic and can hydrolyze a range of different ester bonds, while remaining completely nontoxic to human cells. This catalytic property is maintained even under very harsh conditions, reflecting the very robust nature of the *FapC* amyloid fold.

In summary, our data reveal how the IRs are compatible with heterogeneous cross‐β stacking and provides mechanistic insights into the formation of *FapC* FuA in the *Pseudomonas* extracellular biofilm biogenesis system through straightforward layered stacking of these IRs. Our work provides a strong foundation for the design of novel functionalized biomaterials based on an unusually stable and readily available biological material.

## 2 Results

### 2.1 Sequence Analysis Defines a Shared Motif in the IRs and a Strong Preference for 3 Repeats per *FapC*

**Figure 1:**
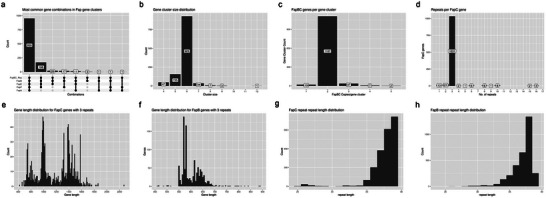
Histograms of the organization of the *Fap* operon in different genomes and the occurrence of imperfect repeats. a) Most common gene combinations in gene clusters. b) Number of genes per cluster. c) Number of *FapBC* genes per cluster. d) Number of imperfect repeats per *FapC* gene. Gene length distribution for genes of e) *FapC* and f) *FapB* with 3 repeats. Domain length distribution of g) *FapC* and h) *FapB*.

To elucidate the distribution in the organization of the *Fap* operon and the number of repeats among organisms producing *FapC* amyloid, we carried out a homology search for the *FapC* repeat sequence as well as the other genes present in the *fap* operon in the Genome Taxonomy Database (GTDB) v214.1. We used our previously developed Hidden Markov Models (HMMs) (*FapA*, *FapBC*_repeat, *FapD*, *FapE*, and *FapF*)^[^ ^25^ ^]^ to perform the search (hmmsearch, HMMer v3.4).^[^ ^25^ ^]^ We filtered our results to only include genes which had *FapBC*_repeat homology and were located in a gene cluster (<5000 bp distance between genes) containing at least 3 other genes with homology to the HMMs. Note that due to the high variability of the nucleator protein *FapB* and the major *Fap* subunit *FapC*, we only had a combined *FapBC*_repeat HMM for these proteins based on their homologous repeat sequences.^[^ ^25^ ^]^ As a consequence, the complete *fap* gene clusters detected in this study contained two distinct genes, both annotated as *FapBC*_repeat. The filtering resulted in 1172 gene clusters, of which 950 (81%) included at least two *fapBC* repeat genes and the remaining 4 *fap* genes (**Figure** **1**a). Within this large group, most clusters (925) contained 6 distinct genes, corresponding to the 6 distinct genes in the *fap* operon (*fapABCDEF*) (Figure 1b). Gene clusters with only 4–5 distinct genes usually lacked the *fapA* gene (5 genes) or both the *fapA* and the *fapF* genes (4 genes). The vast majority (94.5%) of the 1172 gene clusters contained 2 *fapBC* genes (Figure 1c). A similar picture emerges by counting how many *fapBC* genes occur per genome, since 99.5% of all genomes only contained one *fap* operon. Only three genomes contained two *fap* gene clusters. These clusters were never perfect copies of each other, having different gene lengths or different gene compositions. Whole‐operon duplication within the genome and subsequent divergence between the two clusters is more likely than horizontal gene transfer, which is a rare event in the *Fap* system.^[^ ^25^ ^]^

To focus on only *FapC*, we assumed that any gene cluster with two *FapBC*_repeat genes would follow the synteny *fapABCDEF*, designating the first *FapBC*_repeat gene in the clusters as *fapB* and the second one as *FapC*. When we restrict ourselves to *FapC*, we can analyze how many *FapBC*_repeat domains (i.e., number of repeats) we detect per *FapC* gene. Unlike *CsgA*,^[^ ^16^ ^]^ there is a remarkably narrow distribution: out of 1094 *FapC* genes located in *fap* operons with two *FapBC*_repeat genes, 1031 (94.2%) contain 3 IRs (Figure 1d), mostly around 38–39 residues in length (Figure 1g). Note that the *FapBC*_repeat HMM has a length of 39 residues. However, the length distribution for the 3‐repeat genes still varies considerably, showing a multimodal distribution between 700 and 1600 residues with apparent peaks at 750, 1000, and 1400 residues (Figure 1e). For *FapB*, the repeat distribution is as narrow as *FapC*, with 93.9% of 1095 genes having 3 repeats, mostly around 38 residues in length (Figure 1h). The length distribution for the 3‐repeat *FapB* genes is much less varied than for 3‐repeat *FapC* genes, with apparent peaks at lengths of 575 and 640 residues (Figure 1f). The differing gene lengths in *FapB* and *FapC* arise from length differences in the linker regions between repeats, as the repeats have a fixed length (Figure 1g,h).

**Figure 2:**
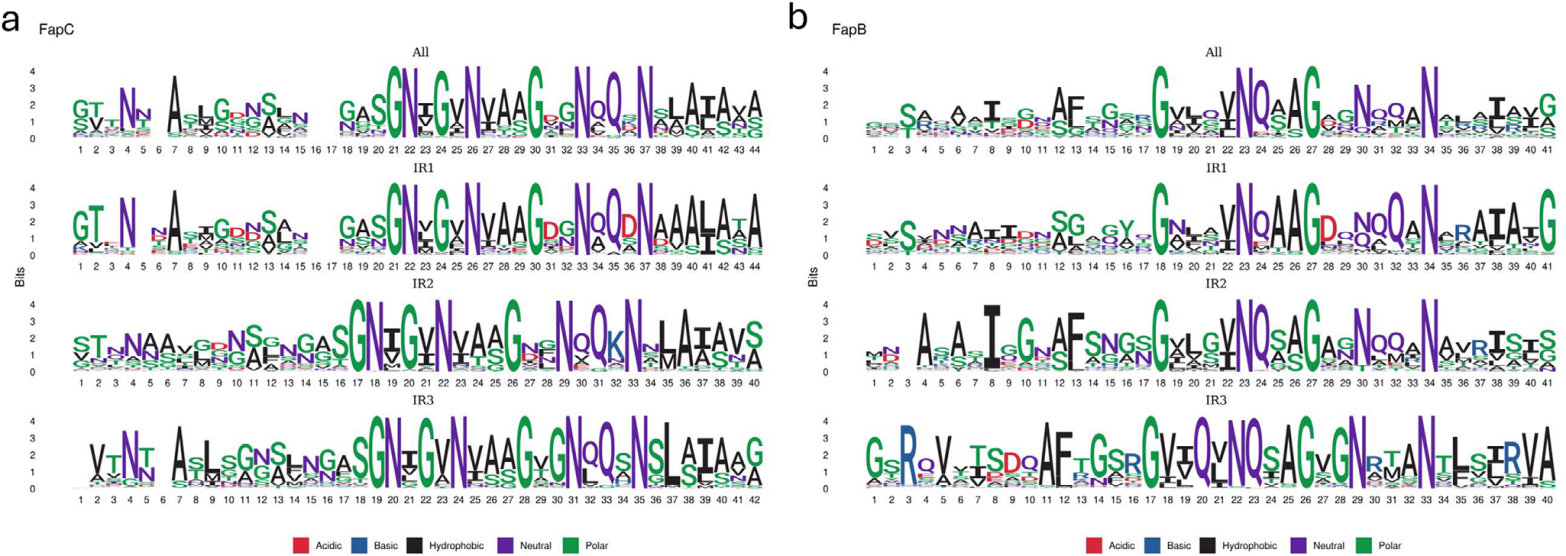
Logos of the most common occurrence of residues in a) *FapC* and b) *FapB* as a function of residue position within the repeat. Small deviations in numbering are due to the occurrence of gaps in some repeats and not others.

Finally, we extracted the sequences of every *FapBC*_repeat hit in the *FapB* and *FapC* genes containing 3 IRs, designating them IR1, IR2, or IR3 in order of location on each gene. We used these sequences to construct sequence logos for each repeat in both *FapB* and *FapC*. Additionally, we created a sequence logo from all the repeat sequences found in *FapB* and a sequence logo from all the repeat sequences found in *FapC* (**Figure** **2**).

The final 27 positions in all three *FapC* repeats are remarkably similar, showing the following motif: gasGNΨGΨNΨaaG*gNqQ*N*Ψ(a/s)Ψ(a/s)*(a/s/g), where small and capital letters denote high and complete conservation levels respectively, Ψ denotes hydrophobic residues, (x/y) denotes equal distributions of residues x and y, and an asterisk denotes no conservation between repeats. While the first 13–17 positions (depending on the lengths of the individual logos) are less similar, they all contain a conserved Asn residue at location 4 and a conserved Ala at position 7 (which corresponds to position 6 in IR2 as it does not have a gap).

The final 24 positions in the *FapB* repeats 1 and 2 are also highly similar and have the consensus motif: G*Ψ*ΨNq*aG**nqq*N***Ψ***, while the final 24 positions of repeat 3 has the consensus motif GΨΨQΨNQ*aG*gN***N*Ψ*Ψ*Ψa. As in the *FapC* gene, there is more variation between the *FapB* repeats in the first 16–17 positions. In general, the variations between *FapC* and *FapB* repeats are greater than the variation within different repeats in *FapB* or *FapC*.

### 2.2 AF3 Prediction of *FapC* Variant Structures Predicts a Greek Key Motif and Multiple Different Conformations among the Linker Regions

Having established the general levels of conservation within the *FapC* and *FapB* repeats, we now turn to a computational and experimental analysis of the structures which these repeats give rise to. We show that the conservation patterns are strongly reflected in these structures. We first predicted the structure of *FapC* fibrils using *FapC* from *Pseudomonas fluorescens* UK4, the shortest variant detected in our computational screening. For this we used AF3,^[^ ^37^ ^]^ whose prediction of *CsgA* fibril structure aligns very well with experimental observations.^[^ ^16^ ^]^ AF3 predicts *FapC* to adopt a Greek‐key‐like motif (Figure S2a, Supporting Information), which is more similar to pathological amyloids than the highly regular β‐helix structure adopted by *CsgA*. Two main cores could be distinguished: i) an inner and partially open core that contains all the conserved residues and a large number of hydrogen bonds between different residue side chains and between side chains and backbones and ii) an outer core with a lower level of conservation and hydrogen‐bond contacts (details in Figure S3 in the Supporting Information). Most of the sequence is integrated into the amyloid structure. The 87 disordered residues (36.6% of the full sequence) comprise the C‐ and N‐termini (residues 1–15 and 214–227) and the links connecting the 3 repeats.

To investigate whether the size of the linkers affects *FapC* structure, we turned to three other 3‐domain *Pseudomona* *s* *FapC* variants, namely *Pseudomonas aeruginosa* PAO1 (340 aa), *Pseudomonas putida* F1 (483 aa), and *P. fluorescens* Pf5 (329 aa). All have linkers longer than those of *FapC* UK4. The length of linker 2 varies considerably (35–259 residues) whereas that of L1 is much more constrained at 28–34 residues (Figure S4, Supporting Information). All 3 variants conserve the amyloid core and the distribution of conserved residues (Figure S2b,c,d, Supporting Information). However, AF3 also predicts with high confidence (except for F1) that the linker regions form β‐strands which interact and thus form an extended amyloid‐like region (Figure S2, Supporting Information). To validate this, we analyzed their aggregation propensity using both linear (Aggrescan) and structural (Aggrescan4D) prediction tools.^[^ ^38^, ^39^ ^]^ As summarized in Figure S5 (Supporting Information), both tools identified aggregation prone regions (APRs) in the core of the amyloid fibril of all the variants. In addition, some short APRs could be also observed in these linkers in PAO1 (175‐YGGTYVSLKLN‐185 and 250‐GTVSGFIPAIVGFKT‐264), F1 (195‐DTITI‐199, 225‐FSVAGA‐230, and 403‐SNTVSFQVLT‐412), and Pf5 (195‐GGFVASGT‐202 and 244‐ELAGYWTQ‐251). Altogether, it seems plausible that these regions are able to form β‐strands, but their role in the fibril structure and formation remain unclear. They could contribute to an extended fibril core, an independent domain outside the main amyloid structure or be involved in lateral interactions between neighboring fibrils, leading to a higher tendency of clustering.

### 2.3 *FapC* UK4 Aggregation Is Promoted by Other *FapC* Strain Variants Corresponding to Their Similarity in Sequence

**Figure 3:**
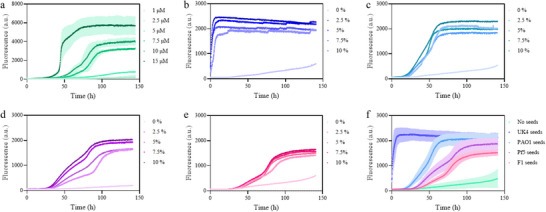
Kinetic time profiles for aggregation of UK4 and with/without different seeds. a) ThT fluorescence intensity of UK4 is plotted over time at initial monomer concentrations ranging from 1 to 15 µm. b–e) Aggregation kinetics of UK4 in the presence of different concentrations of UK4, PAO1, Pf5, and F1 as seeds. 5 µm UK4 monomer was incubated with 0%, 2.5%, 5%, 7.5% or 10% v/v seeds. f) Comparison of seeded fibrillation of 5 µm UK4 monomer with 7.5% seed of the indicated *FapC* strains.

The natural occurrence of different strains of *Pseudomonas* with variable *FapC* length and sequence composition makes it biologically relevant to assess to what extent these strains can reinforce each other's tendency to self‐assemble. We tested this by exposing seeds of sonicated *FapC* fibrils formed by four different *Pseudomonas* strains (besides UK4 also PAO1, F1, and Pf5, whose repeat and linker lengths are shown in Figure S4 in the Supporting Information) to monomeric *FapC* UK4. In the absence of seeds, monomeric *FapC* UK4 fibrillates with the characteristic sigmoidal time curve in a concentration‐dependent fashion (**Figure** **3**a). At 5 µm protein, fibrillation is sufficiently slow that no significant amyloid is formed within the observed 145 h. However, the addition of preformed UK4 seeds accelerates this in a dose‐dependent manner and completely eliminates the lag phase even at the lowest seed concentration of 2.5% (Figure 3b). Seeds of the 3 other fibrils also accelerate the process significantly (Figure 3c–e), but the lag phase remains at all concentrations and with all fibrils. Direct comparison using 7.5% seeds (Figure 3f) shows a clear ranking with UK4 >> PAO1 > Pf5 > F1. Interestingly, the linker regions of PAO1 and Pf5 are comparable in length, while that of F1 is significantly longer (Figure S4, Supporting Information). Strikingly, AmyloFit analysis revealed changes in the aggregation mechanisms depending on the seeds used. When *FapC* UK4 aggregation is induced with seeds from the same UK4 strain, the kinetic process is controlled by multiple secondary processes including fragmentation and secondary nucleation (Figure S6a, Supporting Information). However, when the seeds are produced from different *Pseudomonas* strains, fragmentation becomes the dominant process (Figure S6b–d, Supporting Information) at the expense of other secondary mechanisms. AmyloFit kinetic analysis therefore indicates that the presence of the linkers on the surface of the fibrils limits the seeding process to the elongation of the preformed species, hampering surface‐dependent polymerization. The obtained kinetic parameters (Table S1, Supporting Information) indicate that cross‐seeding also translates into a lower effect nucleation (*k*_n_) and fragmentation (*k*_−_) processes and a higher dependence on the elongation (*k*_+_). Moreover, the depolymerization rate constant (*k*_off_) also suggests a lower stability of the cross‐seeded constructs (Table S1, Supporting Information). Nevertheless, it is important to note that some of the kinetics curves might be suboptimal for a fine‐tuned analysis using AmyloFit.^[^ ^40^ ^]^ This is a result of the complexity of the kinetic behavior, which suggests that multiple microprocesses could be taking place at the same time with no clear dominance of one of them. In this sense, the actual value of the different kinetic constants should not be considered and only consider a simple qualitative comparison of the general pattern changes. In addition, PAO1 seeding and cross‐seeding analysis indicated that only UK4 and PAO1 seeds were capable of promoting fibrillation of monomeric PAO1 (Figure S7, Supporting Information). PAO1 shows sequence homology of 36%, 45%, and 30% with UK4, Pf5, and F1, respectively, making Pf5 the closest homolog of PAO1, but this is clearly not sufficient to promote cross‐seeding.

These observations suggest that while UK4 monomers can nucleate on heterologous fibrils, the efficiency of cross‐seeding is limited by structural incompatibilities between the fibril cores of different variants.

Our bioinformatic analysis identified a small number of *FapC* variants with >3 conserved and IRs. To evaluate the degree of structural conservation of the amyloid core, we selected sequences containing 4 (Figure S8a, Supporting Information), 6 (Figure S8b,c, Supporting Information), 8 (Figure S8d,e, Supporting Information), 10 (Figure S8f, Supporting Information), and 16 (Figure S8g, Supporting Information) repeats from different Gram‐negative bacterial genera (the organisms from which they derive are indicated in the figure legend). Overall, the fibril core is preserved among the different species independent of the number of repeats involved. Also, the location of conserved residues and the high abundance of hydrogen bonding remained unchanged. Nevertheless, there is a clear decrease in the conservation of these residues. This mostly affects the Asn at position 20 or 22 (repeats numbering) and the Gln and Asn at positions 31–35 and 33–37, respectively (conserved residues highlighted in Figure S9 in the Supporting Information). Altogether, the predictions underline the essential role of the conserved repeats in the formation and stabilization of the *FapC* amyloid structure.

The structural predictions revealed that UK4 represents a minimalistic version of the *FapC* family. Therefore, we compared the structural conservation of the UK4 fibril core for variants differing in the number of repeats and the size of the linkers (Figure S10, Supporting Information), for which we obtained a highly confident prediction (Figure S11, Supporting Information). The complete overlap between the predictions, above all in the positions of highly conserved residues, suggests strong evolutionary conservation of the fibril conformation. However, structural homology does not guarantee similar mechanisms of aggregation. For example, the larger size of the linker and/or the increase on the repeat content could potentially alter the primary nucleation or promote a greater impact of secondary processes (i.e., an extended surface could promote secondary nucleation). This is complicated by the fact that the 3 tested *FapC* variants do not bind thioflavin T (ThT) as well as *FapC* UK4, leading to poorly defined fibrillation curves (data not shown). This may in turn be linked to the extended linker regions (evidence for which is presented below) which could block ThT's access to the fibril core.

### 2.4 Cryo‐EM Analysis Identifies Twisted Fibrils of *FapC* UK4 Whose Overall Dimensions Are Confirmed by Atomic Force Microscopy (AFM)

**Figure 4:**
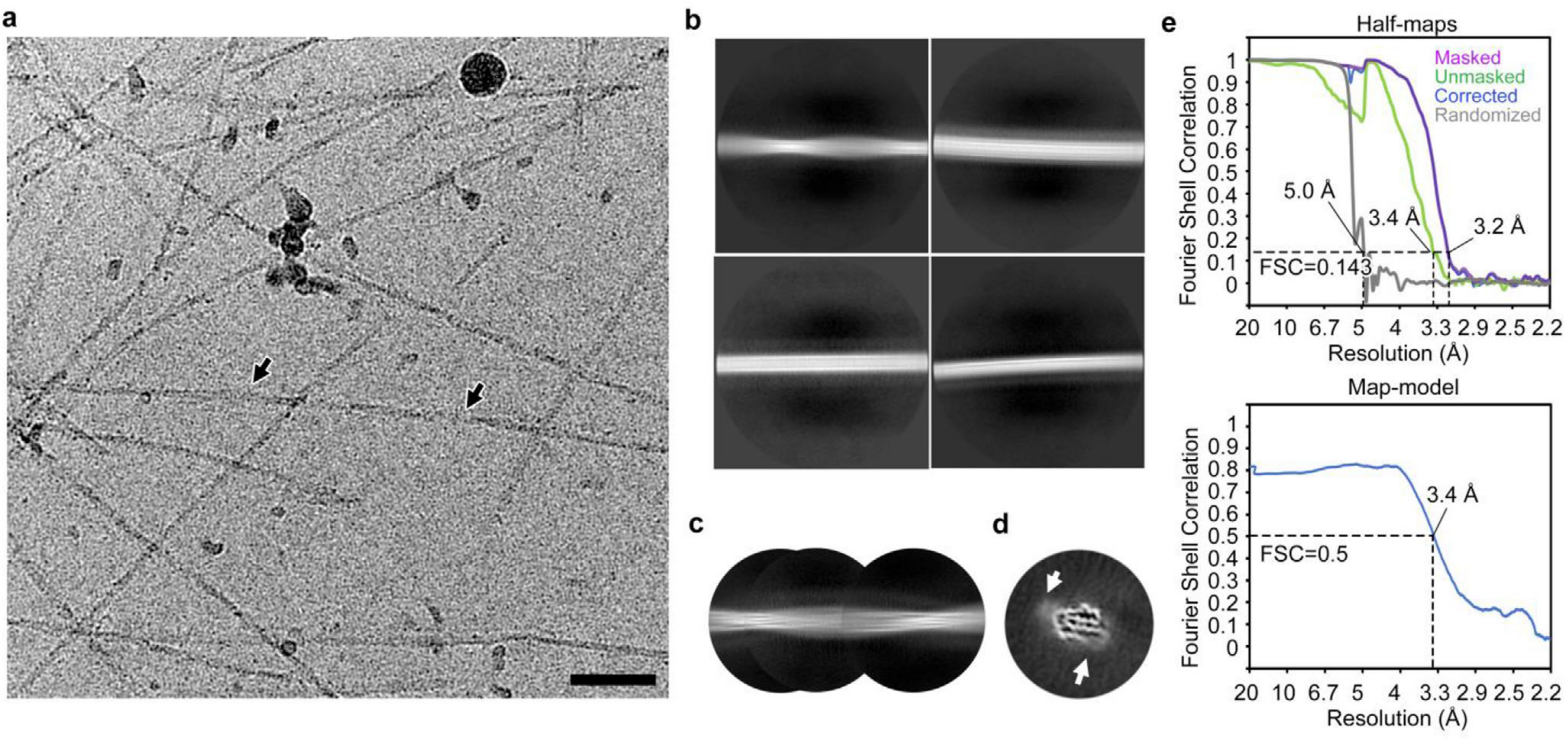
Cryo‐EM data processing of *FapC* fibrils. a) Representative cryo‐EM microscopy image of the collected dataset. Black arrows indicate a twisted *FapC* fibril. Scale bar = 50 nm. b) 2D classes displaying different *FapC* fibril species with a box size of 720 pixels. The top‐left 2D class represents the twisted species. c) A stitched view of the twisted *FapC* fibrils using 2D classes with a box size of 360 pixels. d) 3D reconstruction of the twisted *FapC* fibrils at a resolution of 5 Å. White arrows indicate extra densities around the fibrils, suggesting connections between the main chains in the fibril core. e) FSC curves between two half‐maps (top) and the cryo‐EM reconstruction and refined atomic model (bottom).

**Figure 5:**
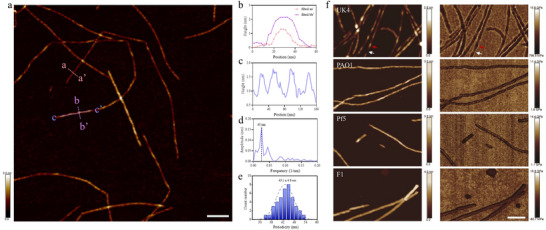
AFM characterization of *FapC* fibrils. a) High‐resolution AFM morphology image of twisted UK4 fibrils. b) Line profiles of two fibrils indicated in (a) along aa′ and bb′. c) Line profile along cc′ in (a). d) Fourier transform analysis of the fibril along cc′. e) Periodic distribution obtained from statistical analysis of 40 fibrils. f) Morphology images (left) and the corresponding stiffness maps (right) of fibrils of UK4, PAO1, Pf5, and F1 from top to bottom. Scale bar in all images is 100 nm.

To validate our computational insights with an experimentally based high‐resolution structure of the *FapC* fibril, we performed an initial negative stain electron microscopy (EM) and cryo‐EM (both in Figure S12, Supporting Information) screen of fibrils of recombinant *FapC* from *Pseudomonas* strains UK4, PAO1, Pf5, F1. Based on this screen, we selected UK4 for high‐resolution cryo‐EM analysis due to its relative high sample quality (**Figure** **4**a). 2D classification resolved two populations: twisted fibrils and nontwisted fibrils of variable widths (Figure 4b,c). We performed 3D helical reconstruction with a selected 2D class of the twist type and the corresponding particles (Figure 4d), achieving a resolution estimated at 3.16 Å with 4.8 Å helical rise, −2° twist, and a ≈864 Å crossover distance for a full 360° turn (Figure S13, Supporting Information). Gratifyingly, this is in excellent agreement with the results of AFM measurements (**Figure** **5**), which we employed to characterize the morphological and mechanical properties of the different *FapC* fibrils. The height image of UK4 (Figure 5a) shows a typical nanofibril network. Because width determination of fibrils by AFM is strongly dependent on the geometry of the AFM tip and AFM image contrast levels, we decided to use the maximum height measurements to characterize the different fibril cross‐sections. Line profiles were extracted along two representative fibrils (Figure 5b), labeled as aa′ and bb′. The height profiles indicate distinct variations in fibril dimensions (≈1.1 or ≈2.2 nm in Figure 5b), suggesting structural heterogeneity within the sample. Further, the taller fibrils show a periodic height fluctuation along the contour of the fibrils. Figure 5c highlights this periodicity along the fibril segment cc′ of a representative UK4 fibril, clearly demonstrating ≈1 nm height variation along the fibril.

The combination of cryo‐EM results and AFM fibril periodicity can be explained by height variations associated with twisting. Fourier transform analysis in Figure 5d quantitatively confirms the presence of this periodic modulation, yielding a twist period of ≈40 nm. Furthermore, statistical analysis of multiple individual fibrils yielded a histogram of periodicity distribution of 43.1 ± 4.8 nm (Figure 5e). Such periodicity corresponds to one half twist, in excellent agreement with cryo‐EM, which provides a 86.4 nm crossover for a full 360° twist (two periods).

### 2.5 AFM Highlights a Mechanically Robust Core and a Flexible Outer Layer Which Varies in Extent among Different *FapC* Strains and Is Confirmed by Small‐Angle X‐Ray Scattering (SAXS) Studies

*FapC*’s twisted structures can also result in mechanical differences within the fibrils, depending on surface contacts. Therefore, we turned to a closer analysis of the fibrils’ material properties. In Figure 5f, AFM height images and stiffness maps of fibrils derived from different *Pseudomonas* strains reveal distinct morphological and mechanical characteristics. UK4 fibrils (Figure 5f, the first row) exhibit a pronounced left‐handed helical twist, consistently observed across multiple fibrils. By contrast, PAO1 (Figure 5f, the 2nd row) and Pf5 (Figure 5f, the 3rd row) occasionally display a twisted morphology, but these occurrences are rare and irregular, indicating that twisting is not a dominant feature in these fibrils. F1 fibrils (Figure 5f, the 4th row) show no observable twist features but are significantly thicker than the other three fibrils.

Stiffness maps further elucidate differences in fibril mechanical properties. UK4 fibrils exhibit significant stiffness variations along their twisted structure. Specifically, regions corresponding to the peaks of the helical twist (marked by red arrows in Figure 5f, first row) exhibit lower stiffness (≈6.8 GPa), whereas the valleys (marked by white arrows in Figure 5f, first row) display higher stiffness (≈9.5 GPa).That is, the twisted (raised) regions of the UK4 fibril are mechanically softer (deforming more easily under force) and more flexible in height compared to the adjacent, lower‐lying regions. IBy contrast, PAO1, Pf5, and F1 fibrils exhibit a characteristic stiffness distribution where the central region appears stiffer than the periphery. This pattern may indicate a mechanically robust core surrounded by a more compliant outer layer. Specially, PAO1, Pf5, and F1 fibrils show no obvious twist and exhibit a relatively uniform stiffness along the central axis, ranging from ≈8.3 to 8.9 GPa, slightly lower than the stiffest regions of UK4 fibrils. However, their peripheral regions are noticeably softer, with stiffness values of ≈5.0 GPa for PAO1, ≈5.2 GPa for Pf5, and ≈3.2 GPa for F1. This stiffness distribution appears to correlate with the length of the *FapC* linker regions (Figure S4, Supporting Information): fibrils with longer linkers exhibit softer peripheral zones, suggesting that extended linker sequences may confer increased flexibility or reduced packing density at the fibril edges.

Given that cryo‐EM does not provide information on flexible regions and our AFM data are based on dried fibrils adsorbed onto mica, we turned to SAXS to obtain low‐resolution information on the full structures. Using a model in which the fibrils are approximated as cylinders allowed us to obtain information on the cross‐section structure of the fibrils in solution. The data, which cover the *q* region that originates from the cross‐section structure, are shown in Figure S14a (Supporting Information) and they display a remarkable similarity, considering the large variation in mass of the *FapC* monomer (UK4 24 kDa, Pf5 32 kDa, PAO1 33 kDa, and F1 48 kDa). The sizes obtained from the cylindrical model are given in Table S2 (Supporting Information). When presenting and discussing the results, it is relevant to consider the mass of the different variants. The fibrils may have a core that is preserved across the variants so the additional mass will impact the total size. The parameters for UK4 corresponds to a cross‐section of 3.4 × 11.3 nm^2^, which is quite large compared to the β‐sheep core part of the AF model (3.7 × 6.5 nm^2^). However, including the loops of the AF model gives a length of 12 nm, which agrees well with the length from SAXS. The Pf5 cross‐section is significantly larger with 5.9 × 18.9 nm^2^. Considering that Pf5 is only 8 kDa larger than UK4, this is quite a large difference. The AF model has a total length of about 20 nm so probably some of the added parts are folding back to give a thicker and slightly shorter size of the cross‐section. PAO1's size of 5.0 × 17 nm^2^ is similar to Pf5, which is reasonable given their similar sizes. For this molecule, the AF model has a length of about 16 nm, agreeing quite well with the size from SAXS. F1 has a cross‐section size of 7.0 × 19 nm^2^, consistent with it being the largest of the 4 variants. As for Pf5 and PAO1, this suggests that the extensions fold back and attach to the fibril surface. The AF model, even neglecting the N terminal loop, has a length of about 24 nm, which also suggests that the added part is folding back in reality. The AF models and the ellipsoids of the cross‐section are displayed in Figure S14a (Supporting Information).

The fits to the SAXS data for the AF atomic models are shown in Figure S14b–e (Supporting Information), where the corresponding models are also shown. The model for UK4 agrees nearly perfectly with the data with *χ* ^2^ = 1.6, whereas the models for PAO1 and Pf5 have some minor systematic deviations, resulting in *χ* ^2^ = 6.0 and 9.3, respectively, although one sees good agreement at low *q*, demonstrating that the cross‐section sizes are well accounted for. The agreement for F1 is qualitatively worse, despite *χ* ^2^ = 5.0, with clear systematic deviations at low *q*, demonstrating that the fibrils are actually smaller in cross‐section size than the AF model predicts. This is also in agreement with the conclusion for the cylinder model, namely that the added parts fold back on the fibril.

In summary, the combination of structural and mechanical differences among these *Pseudomonas* fibrils highlights the diversity in fibril assembly mechanisms, which may influence their functional roles in biofilm formation, surface adhesion, and mechanical stability.

### 2.6 Cryo‐EM Analysis of *FapC* Fibrils at 3.16 Å Resolution Reveals a Greek Key Motif in the Filament Structure and Distinguishes Individual IRs

**Figure 6:**
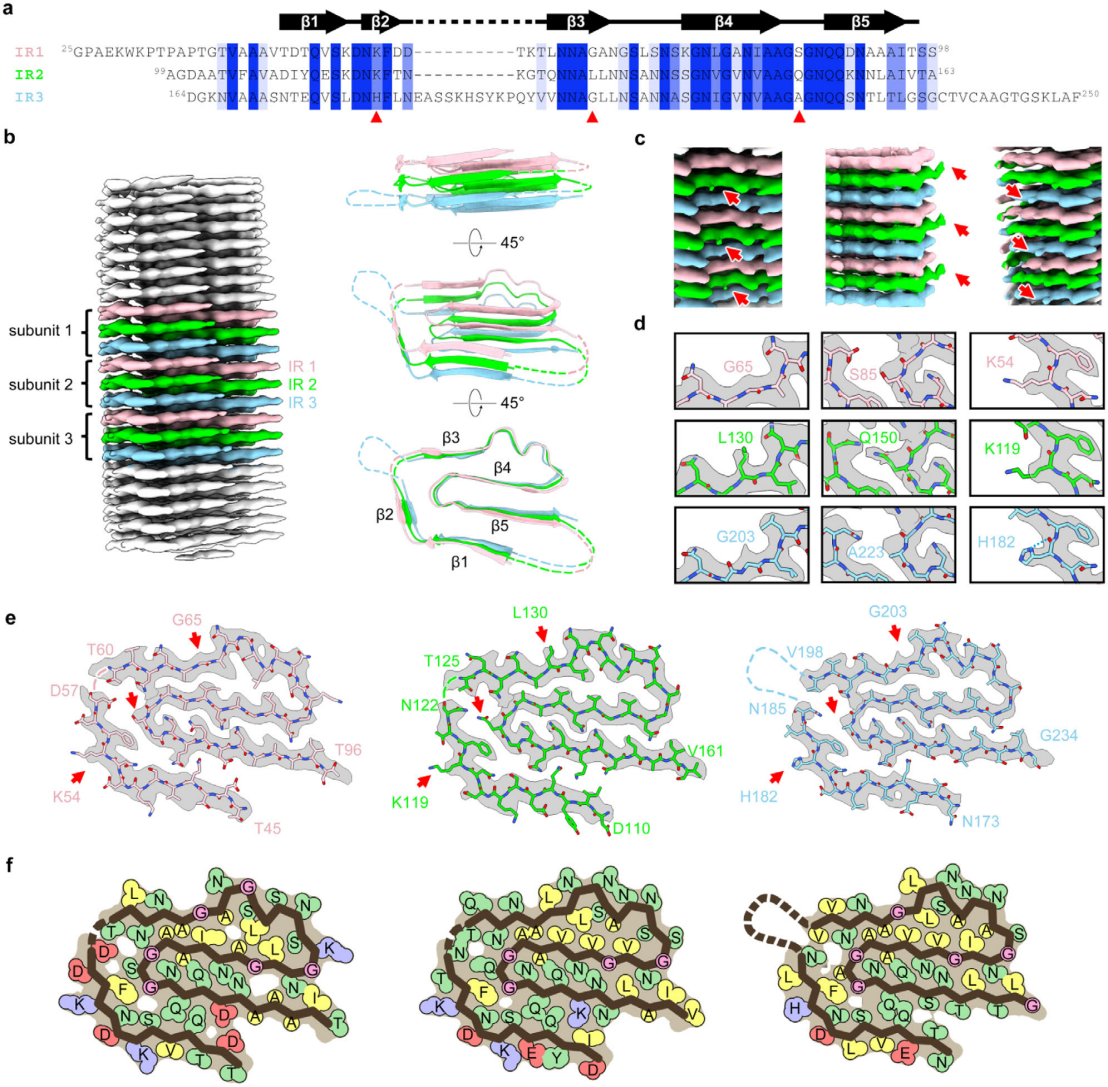
Cryo‐EM structure of *FapC* fibrils. a) Sequence alignment of imperfect repeats shows the conserved residues and secondary structure β strand distribution. Red arrows in this panel and in panels (c–e) indicate the residues whose sidechain electron densities are used to distinguish different imperfect repeats. b) 3D reconstructed electron density map of *FapC* filament with a monomer of three layers as rise unit. c) The map difference of side chains among the three imperfect repeat layers. d) Assignment of side chains according to the difference of map in panel (c) above, also shows the hydrogen bond stabilizing the side chain of H182. e) Fitting models in map (gray) for each imperfect repeat layers, red arrows indicate the position of residues used to distinguish different layers in (c) and (d). f) Residues are classified according to their property: hydrophobic (yellow), uncharged hydrophilic (green), negative charged (red), positive charged (blue), glycine (pink), and also show how they fill in the cavity. White holes between side chains means unfilled space.

Based on our bioinformatic analysis and the AF3 prediction, we expect each *FapC* monomer to form 3 layers of cross‐β structure (one for each imperfect repeat). This makes it inappropriate to average the IRs as perfect repeats (sequences shown in **Figure** **6**a). Indeed, individual IRs could readily be distinguished from the 4.8 Å helical rise map (Figure S13, Supporting Information, see the Experimental Section). We reprocessed the data using a 14.4 Å (3 × 4.8 Å) helical rise and −6° twist, corresponding to a monomer with 3 layers of cross‐β structure. When we performed 3D helical reconstruction, we obtained density maps at 3.2 Å which show great overall similarity between the three layers but with differences in details (**Table** **1** and Figures 4e and 6 and Figures S13 and S15a,b (Supporting Information)). The electron density map shows good connectivity within the backbone α‐carbon chain. We also resolve side‐chain density, with only a very short break corresponding to two amino acid residues (Figure 6b,e). There are extra densities around the fibrils, which we interpret as flexible connections in the form of linker regions between the main chains in the fibril core (Figure 4d, white arrows). The local resolution distributions (Figure S15b, Supporting Information) show that the inner core has the best resolution (<3 Å), in which electron density map for side chains resolved very well, while the predicted flexible region has slightly lower resolution.

**Table 1:** Cryo‐EM data collection, refinement, and validation statistics of *FapC* fibrils.

	*FapC* [EMD‐63855, PDB 9U4U]
Data collection and processing	
Magnification	×130 000
Voltage [kV]	300
Electron exposure [e^−^ Å^−2^]	40
Defocus range [µm]	0.8–3.5
Pixel size [Å]	0.932
Symmetry imposed	C_1_
Helical rise [Å]	14.4
Helical twist [°]	−6.0
Initial particle images (no.)	850 242
Final particle images (no.)	35 507
Map resolution [Å]	3.2
FSC threshold	0.143
Map resolution range [Å]	200–3.2
	
Refinement	
Initial model used (PDB code)	AlphaFold 3 model
Model resolution [Å]	3.4
FSC threshold	0.5
Model resolution range [Å]	200–3.4
Map sharpening *B* factor [Å^2^]	71
Model composition	–
Nonhydrogen atoms	3204
Protein residues	450
Ligands	0
*B* factors [Å^2^]	–
Protein	98.73
Ligand	–
R.m.s. deviations	–
Bond lengths [Å]	0.003
Bond angles [°]	0.592
	
Validation	
MolProbity score	2.30
Clashscore	16.77
Poor rotamers [%]	0
Ramachandran plot	–
Favored [%]	88.89
Allowed [%]	11.11
Disallowed [%]	0

The *FapC* UK4 fibril is made up of a single filament. A model of this *FapC* fibril was constructed by fitting the density map of three layers with the AF3 model which contains three IRs of *FapC* residues 45–96, 110–161, and 173–234 (Figures 4e and 6e), while loop linkers and other flexible region of the protein cannot be modeled due to poor electron density (Figures 4d and 6b,e). Three IRs were distinguished by different electron densities of the side chains between three layers: Leu130 of IR2 has clear side chain density while the corresponding Gly65 of IR1 and Gly203 of IR3 lack the side chain (Figure 6c–e and Figure S13 (Supporting Information)); Gln150 of IR2 has clear side chain density when the cryo‐EM map was shown in lower threshold compared to Ser85 of IR1 and Ala223 of IR3 which have short side chain (Figure 6c–e and Figure S13 (Supporting Information)); His182 of IR3 that form a hydrogen bond with the main chain carboxyl group, which stabilize the conformation of side chain of His182 (a “histidine corner”), is well accommodated within the electron density map, while surface residues Lys54 of IR1 and Lys119 of IR2 have a flexible side chain which usually only show electron density for Cβ (Figure 6c–e and Figure S13 (Supporting Information)), similar to what is seen for Lys residues in numerous high resolution crystal structures of globular proteins. Data collection, processing, and model building statics are listed in Table 1.

**Figure 7:**
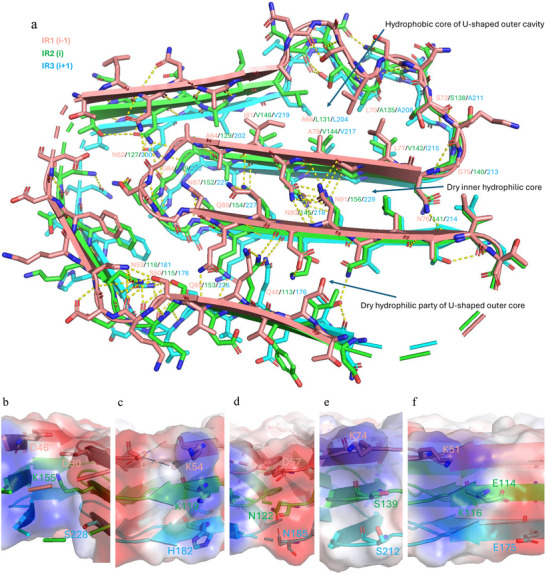
Molecular interactions stabilizing the Greek key‐shaped cross‐β out‐of‐plane staggered structure of *FapC* fibrils. a) Hydrophobic interactions and hydrogen bonds. Molecular interactions stabilizing the Greek key‐shaped cross‐β out‐of‐plane staggered structure of *FapC* fibrils. b–f) Heterogeneous stacking of charged residues.

IRs of *FapC* form a canonical β‐solenoid based on a Greek key arch with a conserved amyloid core. However, in contrast to other amyloids, the three layers of IRs are connected by loop linker sequences, restraining the monomer fold. The Greek key‐shaped cross‐β structure is composed of five short β‐strands, each layer spiraling around the helical axis (Figure 6b). Residues 45–96, 110–161, and 173–234 comprise the *FapC* fibril core of layers 1–3, respectively, excluding disordered residues 58–59 of IR1, 123–124 of IR2, and 186–197 of IR3 which form part of the core but do not show sufficiently high electron density to be mapped on the structure (Figure 6e). The Greek key‐shaped structure is formed with strands β4 and β5 tilting out‐of‐plane relative to β1, β2, and β3 along the fibril axis (Figure 6b). The staggered structure of *FapC* fibril allows a residue from repeat layer (*i*) to interact with the neighboring residues in layers (*i* + 1) and (*i* − 1) within the same β‐sheet, and with residues at the opposite β‐sheet in layer (*i*) and (*i* − 1) or (*i*) and (*i* + 1) to form hydrogen bonding interactions (Figures 6b and **7**a). This out‐of‐plane orientation structure may also favor formation of hydrophobic interactions to form a tightly packed hydrophobic core, where the IRs vary in hydrophobic residues but in a complementary fashion.

### 2.7 *FapC* Has Inner and Outer Hydrophobic Cores Stabilized by Coevolved Residue Packing and Extensive Hydrogen‐Bonding Network

The Greek key arch‐shaped *FapC* monomers form cores inside the monomeric subunits, which compose a U‐shaped outer core with a hydrophobic core extended with a long dry hydrophilic moiety and a dry inner hydrophilic core (Figures 6b,f and 7a). Other parts of the monomer are flexible and do not show visible densities. The three layers of cross‐β structure per monomer means that different amino acid residues make up the core in different layers. Importantly, variations in residues of core between IRs are matched structurally to maintain a well‐packed core within each repeat and a conserved backbone topology, consistent with the principle of coevolutionary conservation.^[^ ^30^ ^]^ However, the match is also imperfect and varies between layers, which could lead to differences in stability and the ability to nucleate during the folding of different layers. For example, Leu204, Ala208, Ala211, Ile215, Val217, and Val219 are well packed in the hydrophobic core of IR3, while Ala211 is replaced with a polar Ser138, and Ile215 is replaced with Val142, resulting in poor packing in IR2. Leu131/204 and Val144/217 are replaced with Ala66 and Ala79, respectively, leaving an unfilled hole in the hydrophobic core of IR1 (Figure 6f). The hole close to the 12 residues loop between Asn185 and Val198 in layer 3 is not well packed; this is also the case in the other two layers. There is also an unfilled hydrophilic hole close to Gly86/151/224 in all three layers. These variations in core residues also facilitate hydrophobic interactions between layers in a way similar to global protein due to the cross‐β out‐of‐plane staggered structure. There are 11 hydrophobic residues in the outer core of IR3, while there are 8 hydrophobic residues in IR1 and IR2. The highly conserved hydrophilic inner cavities are well packed, apart from IR1 where an Ala (position 93) replaces the Leu found at position 158 and 231 in IR2 and IR3. The charged Lys and Glu residues and other uncharged polar residues on the surface are not conserved among these three layers (Figures 2, 6, and 7b–f). This may reflect different roles in the higher‐order organization of *FapC* fibrils, showing a lateral association into thicker bundles as observed in our initial screens for cryo‐EM analysis, and interacting with the surrounding water or other components of biofilm.

In addition to the many hydrophobic interaction in the core, the *FapC* fibril structure is stabilized by an extensive network of hydrogen bonds (Figure 7a). Regular alternating hydrogen bonds form between the main chain of all in‐register parallel β‐strands, stabilizing the β‐sheet along the fibril axis. The polar side chains in the core also form main chain–side chain and side chain–side chain hydrogen bonds within the layer or neighbor layers, which play an important role in shaping the monomer layers. For example, Asn53/118/181, Ser50/115/178, and Asn62/127/200 form hydrogen bonding networks in the turns stabilizing β‐arcs. More strikingly, the five highly conserved Asn/Gln polar residues located inside the inner core engage in extensive side chain hydrogen‐bonding in the inner core of the Greek key arch. These form β‐arcs with inward facing compact steric zipper residues between them, which bend the outer longer β‐arch to form the Greek key arch structure. The cryo‐EM structure suggests that Asn and Gln residues maintain the shape of the Greek key arch in the *FapC* fibril, which may explain their high conservation in the predicted structures of *FapC* family. Moreover, heterogeneous stacking of IRs also favors complementary charge interactions between layers to stabilize the monomer fold (Figure 7b–f). Examples include Lys155 on IR2 with Asp46 and Asp90 on IR1; Asp56 on IR1 with Lys54, Lys119, and His182 stacking in the same position in IR1‐3; Glu114 on IR2 with Lys51 (IR1) and Lys116 (IR2). In other cases, heterogeneous stacking avoids the direct charge repulsion that would result from homogeneous stacking as seen for single‐layer monomers forming pathological fibrils. For example, Asp57 is separated by Asn122 and Asn185, while Lys74 is separated by Ser139 and Ser212 as is Glu175 from IR3.

### 2.8 The Cryo‐EM Structure Shows Good Correspondence with AF3 but with Differences in β‐Strand Planes

**Figure 8:**
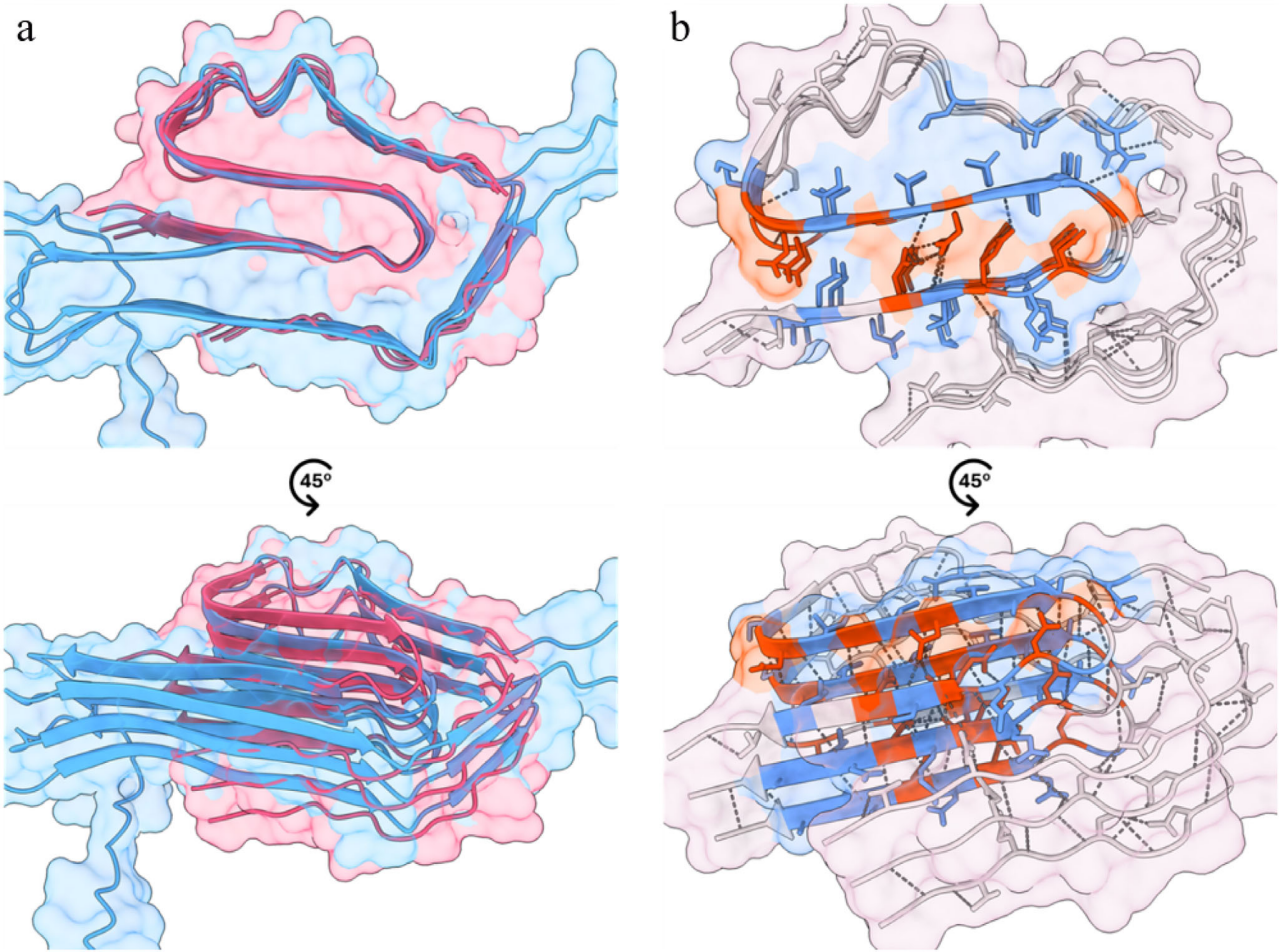
Analysis of the structure of *FapC* UK4. a) Structural homology between AF3‐predicted (blue) and cryo‐EM‐described (pink) *FapC* structure. b) Structural representation of *FapC* sequence conservation; highly conserved residues are shown in red, mid‐to‐low conserved residues are shown in blue, and nonconserved residues are shown in light gray; hydrogen bonds are shown as dark gray dotted lines.

Remarkably, the AF3 prediction of the *FapC* structure exhibited an impressive correspondence with the actual cryo‐EM structure (**Figure** **8**a), and only a few residues predicted to form β‐strands are missing in the characterized structure. Just as predicted, highly conserved residues (Figure 2) are indeed located and oriented to the inner core, while low conserved residues are mostly oriented toward the external core and nonconserved faces the fibril surface (Figure 8b). Moreover, these high‐ and medium‐conserved residues establish most of the hydrogen bonds within the same repeat and perpendicular to the axis between the different repeats. It is evident that these residues play an essential role in fibril formation and stability. AF3 predicted that the β‐strands of the same repeat are on the plane, but the β‐strands tilted out of the plane in the actual cryo‐EM structure. This staggered structure facilitates hydrogen bonds between different repeats and hydrophobic interactions within the hydrophobic core as described above. Thus, the actual structure of the *FapC* fibril shows a more globular‐like diversity in packing than the very locally based packing in the predicted structure of the *FapC* monomer.

### 2.9 Biophysical Characterization of the *FapC* Fibrillation Process prior to Mutagenesis

**Figure 9:**
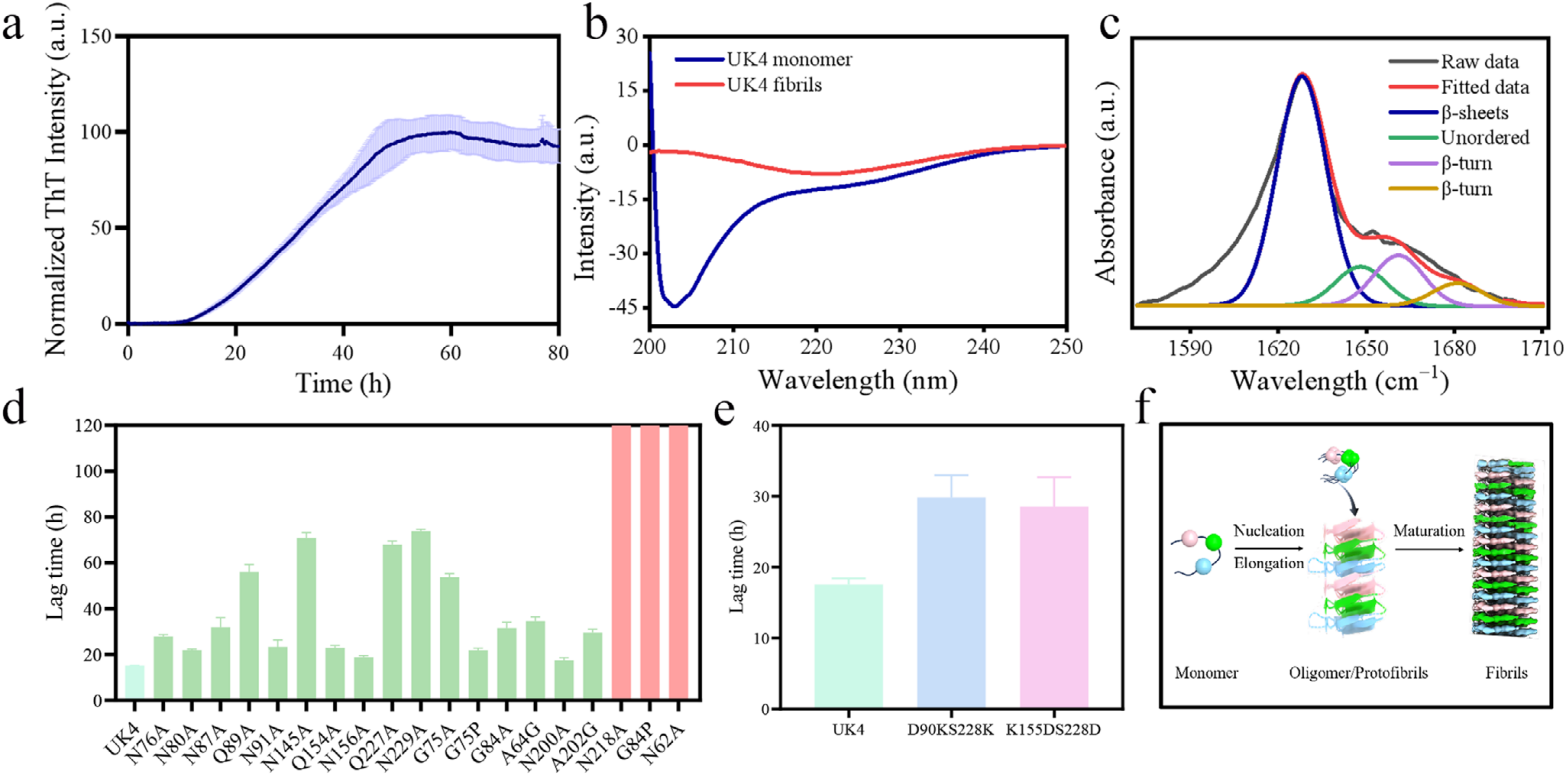
a) Amyloid fibril formation of UK4 monitored by ThT fluorescence measurement. b) CD spectra of freshly prepared monomeric UK4 sample, and after incubating at 37 °C for 72 h fibrillar UK4 sample. c) Secondary structure characterization reported by ATR‐FTIR absorbance spectra (Amide I region) of 37 °C, 168 h incubated UK4 fibrillar sample. d) Effect of mutations of conserved N/Q/G on fibrillation lag time, the red column means no fibril form within 120 h. e) Charge mutation effect on fibrillation lag time. f) Schematic representation of UK4 fibril formation.

The cryo‐EM structure immediately suggests mutagenesis of various centrally placed residues (see below). To provide a basis for comparison with these *FapC* mutants, we performed a comprehensive characterization of *FapC* UK4 wildtype (wt)hy amyloid fibril formation through multiple biophysical techniques, which builds on our extensive previous analysis of its aggregation mechanism.^[^ ^33^ ^]^ ThT fluorescence kinetics reveal the characteristic sigmoidal aggregation curve, in which a pronounced increase in fluorescence intensity occurs after ≈15 h (**Figure** **9**a), indicative of fibril nucleation and subsequent elongation, followed by a plateau phase marking fibril maturation, from where the cryo‐EM sample was taken. Circular dichroism (CD) spectroscopy further corroborates this structural transition (Figure 9b), where the freshly prepared UK4 monomer exhibits a pronounced negative ellipticity near 215 nm, characteristic of disordered or α‐helical structures (Table S3, Supporting Information). By contrast, the fibrillar form, obtained after 72 h of incubation at 37 °C, exhibits a diminished signal, reflecting a conformational shift toward a β‐sheet‐rich architecture (Table S3, Supporting Information). Attenuated total reflectance Fourier transform infrared (ATR‐FTIR) spectroscopy provides further secondary structure insights (Figure 9c), where deconvolution of the amide I region reveals a dominant β‐sheet contribution (≈1620 cm⁻¹), a hallmark of amyloid fibrils, along with minor contributions from unordered and β‐turn structures (Table S4, Supporting Information).

### 2.10 Mutagenic Analysis of *FapC* Structure Confirms Central Role of Conserved Residues in the Fibrillation Mechanism and in Fibril Integrity

After inspecting the cryo‐EM structure of UK4 *FapC* in light of the logo sequence (Figures 2 and 8), we introduced mutations to Ala of the conserved non‐Gly residues in the logo (4 Asn and 1 Gln) to test their contribution to fibrillation kinetics (representative ThT curves shown in Figure S16 in the Supporting Information). We find that all mutations slow down the fibrillation process (Figure 9d), indicating that hydrogen bonds formed by them are contributing to the process of fibrillation. Asn87 and Gln89 located in the most inside of the inner core are the most important in IR1, with the lag time increasing significantly upon mutation (Figures 7a and 9d). For mutations in IR3, the lag times of Gln227Ala and Asn229Ala also increase significantly (Figures 7a and 9d), while Asn218Ala does not fibrillate at all within the measuring time. This indicates that IR3 may be the most important IR among these three IRs in the aggregation process, which is also reflected in its superior level of packing with minimal cavities compared to IR1 and IR2 (Figure 6f). The role of the conserved Gly residues in each repeat is the best probed by a Gly‐to‐Ala mutation, since this reduces backbone flexibility without introducing too many new interactions. In fact, Gly75Ala and Gly84Ala slow down the fibrillation process (Figures 7a and 9d). Gly‐to‐Pro is a more extreme mutation which enormously rigidifies the backbone. Thus, Gly84Pro was completely unable to fibrillate within 120 h (Figure S17, Supporting Information), while Gly75Pro is still able to form fibrils but with increased lag time (Figure 9d). The different effects of the two mutations may reflect that different backbone flexibility is required in different positions of *FapC* during fibrillation. The conserved residues outside the central core of *FapC* are also important for the fibrillation, with an increased lag time of Asn200Ala and no detectable fibrillation of Asn62Ala. Residue 62 is located in the turn of the outer core, highlighting the importance of its hydrogen bond network (Figures 7a and 9d), while the conserved hydrophobic residues Ala64 and Ala202 show their contribution in nucleation (Figures 7a and 9d).

To confirm the advantage of heterogeneous stacking of IRs in balancing charge interactions between layers, at the position around Lys 155 (Figure 7b), we introduced negative charge repulsion by the mutations Lys155Asp and Ser228Asp, resulted in a cluster of four negative charges (Asp46, Asp90, Asp155, and Asp228). Similarly, we introduced positive charge repulsion by mutations Asp90Lys and Ser228Lys, resulted in a cluster of three positive charges (Lys90, Lys155, and Lys228). Both these clusters increased the lag time of fibrillation significantly (Figure 9e) but still formed fibrils (Figure S18, Supporting Information).

We used the webserver AmyloFit^[^ ^40^ ^]^ to determine the effect of 3 different mutations (N145A, Q89A, and G84A) on the *FapC* aggregation process. The selected mutations increased the lag time of fibrillation to different extents (Figure 9d), but kinetic analysis revealed that all of the polymerization processes were dominated by secondary nucleation mechanisms rather than nucleation–elongation (Figure S19, Supporting Information). Strikingly, all the aggregation kinetics of our selected mutants were fitted better by fragmentation‐dominated models than the wt *FapC* variant (Figure S19, Supporting Information). This rise in fragmentation tendency suggests that the mutated fibrils are indeed less stable than the wt counterpart, as suggested by our arbitrary score, and demonstrates the evolutionary optimization of the amino acid composition.

Based on our bioinformatic analysis, AF3 prediction, biophysical characterization, mutation analysis, and cryo‐EM structure, we present a schematic representation illustrating the fibrillation pathway, highlighting key stages including monomeric assembly, oligomer/protofibril formation, and eventual maturation into well‐ordered fibrillar aggregates (Figure 9f). These findings collectively confirm that UK4 undergoes a conformational transition from a disordered monomeric state to a β‐sheet‐rich amyloid fibrillar structure, consistent with the molecular mechanisms underlying amyloidogenesis.

### 2.11 The *FapC* Amyloid Fold Shows Great Promise as a Functional Scaffold for New Biomaterials: Intrinsic Catalytic Properties, High Structural Stability, and Lack of Cytotoxicity

**Figure 10:**
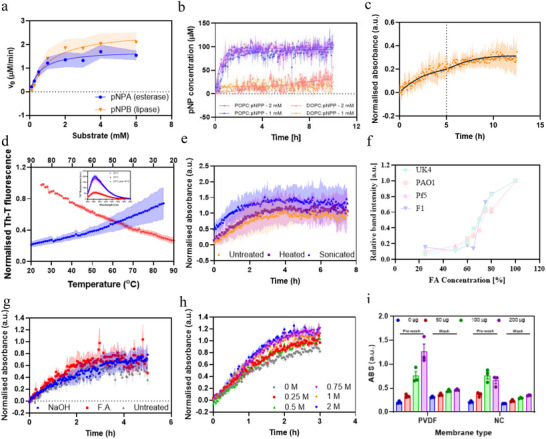
Catalytic potential of *FapC* fibrils. a) Michaelis–Menten analysis of the esterase and lipase capacity of *FapC* fibrils against *p*NPA and *p*NPB. b) Lipase activity against POCP and DOPC vesicles reported upon *p*NPP degradation. c) Recycling of the catalytic activity of *FapC* fibrils upon the addition of fresh *p*NPA after 5 h reaction. d) Fibril stability upon temperature changes reporter by ThT fluorescence; inset shows the final point at 25, 90, and 25 °C after cooling. e) Impact of harsh conditions on *FapC* fibrils’ esterase activity using *p*NPA. f) Monomer release upon FA incubation of different *FapC* fibrils. g) Extreme pH (3 m NaOH or 20% FA) and h) ionic strength (0–2 m NaCl) have very little impact on *FapC* catalytic activity toward *p*NPA. i) Catalytic capacity (hydrolysis of *p*NPA) of *FapC*‐functionalized membranes.

Having presented the structure of the *FapC* amyloid state and the process of its formation, we now turn to the material properties of this amyloid state, which turn out to be exceptional in several ways. Previous studies have shown that pathological amyloids and fibrils of short peptides such as glucagon and *Staphylococcus aureus* PSM peptides have catalytic properties, particularly facilitating hydrolytic processes such as cleavage of ester and amide bonds.^[^ ^8^, ^9^, ^10^, ^11^ ^]^ However, there are no reports of this for bacterial FuA such as *FapC*. We therefore evaluated whether *FapC* UK4 fibrils harbored catalytic activity. Strikingly, incubation of 10 µm *FapC* fibrils with the substrates *para*‐nitrophenyl acetate (*p*NPA) and *para*‐nitrophenyl butyrate (*p*NPB) led to a significant formation of the product *p*NP (**Figure** **10**a) at rates similar to or higher than previously described fibrils (Figure S20, Supporting Information).^[^ ^8^, ^9^, ^10^, ^11^ ^]^ Lipolytic activity was tested using *p*NP‐palmitate (*p*NPP), which *FapC* fibrils were able to degrade when embedded in POPC‐based vesicles, but not DOPC‐based vesicles (Figure 10b), suggesting catalytic specificity. While the solution of *p*NPP became visibly yellow, incubation of *FapC* fibrils with soluble or vesicle‐embedded *p*NPP led to coprecipitation of the fibrils and the lipidic content, which might contribute to part of the observed absorbance increase. *FapC* fibrils also showed a modest recycling capacity upon addition of fresh substrate (Figure 10c), suggesting that *FapC* could be exploited as a reusable biomaterial.

We also explored the possibility of functionalizing surfaces with *FapC*, inspired by reports on the use of Sup35 fibrils to act as antiviral agents when fused with SARS‐receptor binding domains when deposited on polyvinylidene fluoride (PVDF) surfaces.^[^ ^41^ ^]^ We functionalized the surface of PVDF and nitrocellulose (NC) membranes with different amounts of *FapC* fibrils and tested whether they preserved their catalytic properties. Initially, *FapC*‐functionalized membranes exhibited a large hydrolytic potential (Figure 10i). This capacity was reduced, but still significant, after washing the membranes, either due to the release of part of the fibrils from the membrane or to its modest recycling properties. While requiring an optimization of the immobilization process, these results suggest that *FapC* fibrils could be employed to functionalize different surfaces with the desired properties.

Although the values of the catalytic constants indicate that unmodified amyloid fibrils have low efficiency as enzymes, their general robustness and resistance to harsh conditions make them promising scaffolds to develop functional materials. To demonstrate this, we first evaluated the resistance of *FapC* fibrils to high temperature, following ThT changes depending on the temperature. There was a progressive decline in ThT emission with increasing temperature which could be completely recovered by decreasing the temperature (Figure 10d). This suggests that the fluorescence decrease is associated to an effect on the ThT molecule rather than on the fibril stability. This interpretation was confirmed by recording far‐UV CD spectra of the fibrils before, during and after heating, which confirmed the retention of the original amyloid structure (Figure S22, Supporting Information). The esterase activity of fibrils incubated at 95 °C demonstrated that the catalytic potential remained unaltered (Figure 10e). In addition, functional materials should be also resistant to mechanical stress that can alter their physical properties. When *FapC* fibrils were sonicated into smaller fragments, their esterase activity was not only retained but slightly increased (Figure 10e). Another stress factor is extreme pH. Also here *FapC* fibrils excel. All 4 *FapC* variants (UK4/PAO1/Pf5/F1) retain high and similar resistance to dissociation in formic acid (FA), as quantified by sodium dodecyl sulfate–polyacrylamide gel electrophoresis (SDS‐PAGE) (Figure 10f and Figure S21a (Supporting Information)), with ≈70% FA required to dissolve half of the fiber population. The same FA analysis also revealed that *FapC* UK4 mutations only altered the kinetics of fibrillation but not its stability once formed (Figure S21b, Supporting Information). Additionally, ThT fluorescence analysis demonstrated that the fibril conformation is not altered after incubation under high alkaline (NaOH) or acidic (FA) conditions (Figure S21c, Supporting Information) and esterase studies confirmed retention of activity even after incubation in 3 m NaOH or 20% FA (Figure 10g). Unsurprisingly, changes in ionic strength also have very modest effects on *FapC* hydrolytic properties (Figure 10h). Thus, we conclude that the functional amyloids not only display an elegant and highly robust design but have intrinsic catalytic affinity. Added to this attractive property, *FapC* fibrils do not show any cytotoxic activity toward human cells such as HEK293 when tested in Reactive Oxygen Species (ROS) assays (Figure S23, Supporting Information). Accordingly, we propose that the functional amyloid scaffold is an excellent starting material for the engineering of new functional material, e.g., by grafting enzymatic active sites into the amyloid backbone.^[^ ^7^ ^]^

## 3 Discussion

### 3.1 Assembly Mechanism of Cross‐β Stacking with IRs of FuA

The number of determined amyloid structures has increased significantly in the last decade, revealing a very extensive polymorphism among pathological amyloid fibrils. It is to be expected that FuA will show much less variability in structure, since it has been under evolutionary pressure to develop and maintain a robustly reproducible amyloid structure. The most prominent functional bacterial amyloids, i.e., *CsgA* and *FapC*, have multiple imperfect repeats within each polypeptide chain, raising the question of how these IRs are accommodated within the amyloid structure, since they will lead to heterogeneous cross‐β stacking between the different layers. By contrast, the much smaller FuA peptides PSMα1 and PSMα4 do not have IRs and their structures are similar to the classic cross‐β structure of pathological amyloid protein.^[^ ^42^ ^]^ Frustratingly, the best resolution of the FuA protein *CsgA* structure with imperfect repeat sequences is currently 4–6 Å, which is not enough to provide details of the side chains and interface between stacked monomers.

Here, we provide the first high‐resolution structure for an amyloid fibril from the *FapC* family. *FapC* from *Pseudomonas* sp. UK4 represents the simplest *FapC* amyloid version with relatively short linkers and the canonical three IRs and is thus an appropriate first step in our elucidation of the design principles of this family. Its structure immediately reveals important design principles and clues for the assembly mechanism using cross‐β stacking with IRs of FuA proteins. First, a judicious combination of Gln/Asn/Gly residues provides important structural guidance. Gln/Asn forms an extensive network of hydrogen bonds with side chains and backbones in the core, turns, and between layers to stabilize the overall architecture of each IP layer, while flexible Gly residues allow the backbone to twist sufficiently to underpin the conserved Gln/Asn connections. The motif is maintained even in the presence of unfilled cavities in the core. Second, this Gln/Asn framework is supported by hydrophobic residues within the IRs varying in a complementary fashion to form a tightly packed hydrophobic core without changing in the conserved backbone topology. Third, charged residues and other uncharged polar residues on the surface are not conserved among these three layers. This heterogeneous stacking of different and complementarily charged IRs avoids charge repulsion between layers to stabilize the monomer fold. This is in stark contrast to the homogeneous stacking in pathological amyloid which destabilizes the fibril structure. Furthermore, this type of packing may facilitate higher‐order organization of *FapC* fibrils and interactions with the surrounding components of biofilm. In addition, the cross‐β out‐of‐plane staggered structure may also favor formation of hydrophobic interactions and hydrogen bonds between layers, packing the protein monomer in a more globular way.

In addition, our cryo‐EM structure identifies hydrogen bonds involving moderately conserved residues that could play a role in the stabilization of the fibril surface upon the formation of some H‐bonds with the conserved amyloid core. Several H‐bonds are also observed connecting the different backbones of the poorly conserved regions, suggesting that they contribute to the overall stability of the fibril.

### 3.2 Implications of the *FapC* Structure for Biofilm Biogenesis

The interplay between the different IRs is a critically defining feature for FuA, strongly imprinted in the sequence conservation and manifested in our presented structure. All the highly conserved residues identified in our sequence comparison are involved in extensive sidechain hydrogen bonding networks, stabilizing the characteristic Greek key architecture of the *FapC* fold and explaining how *FapC* favors a uniquely well‐defined monomorphic amyloid structure. Sequence comparisons reveal that the *FapC* version with 3 IRs is overwhelmingly the most prevalent form, suggesting that this number of repeats is optimal for self‐assembly and stability and directs a simple consecutive self‐assembly pathway in which the stacking of a small number of similar but distinct layers lead to a uniform directionality in fibril growth. We have previously shown that stepwise removal of these repeats lowers the stability of the ensuing fibrils and makes them more prone to fragmentation during the self‐assembly process.^[^ ^33^ ^]^ Given that *FapC* increases the mechanical strength of the bacterial biofilm,^[^ ^43^ ^]^ we speculate that this is based on physical entanglement of highly stable individual *FapC* fibrils. While we do not yet have high‐resolution structures of other *FapC* variants with longer linker regions, the ability of different *FapC* strains to cross‐seed each other to a significant extent suggests a fundamental similarity in fibril architecture and an ability to accommodate variations in linker regions, perhaps as flexible “fuzzy coats” around the main amyloid core. It also opens up for the possibility of interspecies collaboration within different bacterial strains producing variants of *FapC* and pooling their monomeric *FapC* for mutual fibrillation, though the extent of cross‐talk will be limited by the degree of similarity between the species.

The *FapC* structure also provides insight into the role of the different repeats on the aggregation process and, potentially, fibril stability. The higher impact of the mutations located in the core of the IR3 in the lag phase and extent of fibril formation suggest a key role for IR3 in fibril elongation. By contrast, the lowest impact of IR2 suggests a greater effect on fibril stability than on the aggregation process. IR1 mutations have an intermediate effect, suggesting a minor effect on the aggregation process but greater importance with regards to stability. We also find it noteworthy that the highest impact of the mutations was observed within the loops of the IR1. We speculate that this reflects the need to provide a certain level of flexibility to facilitate the formation of the Greek‐key motif in the IR1 to accommodate the monomer on the surface of the already folded IR3.

### 3.3 Biological FuA Variation Highlights Material Consequences of Different Linker Lengths

The present study has provided the atomic structure of what may be considered the minimal version of the *FapC* amyloid fold, namely that of the UK4 variant. The 3 variants from other *Pseudomonas* species have significantly longer linker regions and have so far defied high‐resolution analysis, though efforts are ongoing for these and other variants. However, the existence of a wide range of naturally occurring variants of *FapC* provides a great opportunity to explore the biologically driven diversity in fibril properties. By complementing our cryo‐EM work with AFM and SAXS on all 4 *FapC* variants, we have shown that increased linker length generally leads to a nanomechanical softening of the fibrils. However, the linkers are not necessarily completely dynamic, since our SAXS modeling suggests a looping back of these segments onto the fibril core. This is consistent with AF3 predictions which highlight different levels of organized structure which extend the amyloid core of *FapC*.

These differences in fibril morphology and mechanical profiles can be partially attributed to sequence variation in the *FapC* proteins, particularly in the lengths of their intrinsically disordered linker regions. As shown in Figure S4 (Supporting Information), the linker regions L2 vary significantly among strains: UK4 (35 residues), Pf5 (103 residues), PAO1 (119 residues), and F1 (259 residues). These differences correlate with fibril architecture and mechanical features. UK4 fibrils, with the shortest linker, exhibit a well‐defined twisted morphology and marked stiffness heterogeneity. By contrast, F1 fibrils, with the longest linker, display a nontwisted morphology and a pronounced core–shell stiffness gradient.

This trend suggests that extended linker regions confer increased flexibility or reduced packing density in the fibril periphery, thereby affecting both morphology and mechanical integrity. These findings align with recent studies showing that local structural flexibility, often imparted by disordered regions such as linkers, plays a crucial role in polymorphic or adaptive self‐assembly mechanisms.^[^ ^44^, ^45^ ^]^ In the case of *FapC* fibrils, longer linkers likely increase local conformational freedom during assembly, resulting in fibril architectures with diminished twist and softer peripheries. This suggests that the linker domains are not merely passive spacers, but contribute actively to the physicochemical tuning of the amyloid structures, enhancing the diversity and functionality of the assembled materials.

Interestingly, AFM imaging and AF3 structural predictions show strong concordance in revealing the structural and mechanical diversity among *FapC* fibrils from different *Pseudomonas* strains. UK4 fibrils exhibit pronounced twisting and periodic height modulations, consistent with the tightly packed and helical β‐sheet architecture predicted by AF3. By contrast, PAO1, Pf5, and F1 fibrils appear more linear and lack periodicity, reflecting looser or asymmetric β‐sheet arrangements in their predicted structure surfaces than in the fibril core. Quantitative nanomechanical mapping further supports these differences, with UK4 fibrils displaying significantly higher stiffness compared to the softer fibrils from other variants. It is worth noting that AF3 tends to predict a β‐sheet stacking of the linker regions, a tendency that complicates the structural prediction of larger complexes. However, this laterally extended stacking, which one would expect to translate into a rigid conformation or lateral interactions, presents as a general trend a lower confidence than the core of the dimer (Figure S11, Supporting Information) and, therefore, other arrangements could be expected. Accordingly, the lower stiffness of PAO1, Pf5, and F1 fibrils measured by AFM under our specific working conditions correlates with this lack of predicted confidence. Altogether, these results demonstrate that the polymorphism of *FapC* fibrils arises from intrinsic differences in β‐sheet packing and supramolecular organization, highlighting the power of combining AFM with structure prediction tools to dissect the architecture of functional amyloids but at the same time highlighting a need for improvement in the ability to predict structures of complex regions.

Future structural and nanomaterial investigations of these variants will likely lead to further insights into their functional roles in biofilm formation, surface adhesion, and mechanical stability.

### 3.4 *FapC* as a Functional Scaffold for Biomaterials

The mobilization of amyloid fibrils as potential biomaterials has raised great interest due to their exceptional structural properties. Multiple applications have been extensively explored, fusion constructs of the amyloid component with a globular enzyme.^[^ ^6^ ^]^ Yet, the intrinsic catalytic properties of the archetypal functional amyloid fibrils *CsgA* and *FapC* remain strangely unexploited. Here, we demonstrate that *FapC* fibrils hold great potential as scaffold for developing new materials. The high level of compactness and the dense H‐bond network revealed by the cryo‐EM structure translates into an exceptionally stable architecture, resisting high temperatures and extreme pH conditions. The fibrils perform multiple hydrolytic processes including esterase and lipase, and these properties are maintained after incubation under harsh conditions. Furthermore, the fibers demonstrated a highly specific activity as shown by the preference of POPC over DOPC vesicles, possibly related to DOPC's lower level of saturation and the more conical conformation compared to POPC. Whether this catalytic behavior is related to an evolutionary constraint or to a random consequence of the monomer stacking on the fibril conformation requires further studies and engineering efforts.^[^ ^7^ ^]^

Altogether, these results provide a general framework for developing therapeutics against antimicrobial resistance and designing *FapC*‐derived functional biomaterial as suggested by us previously,^[^ ^6^ ^]^ more specifically involving the redesign of *FapC* fibril to perform a desired function. As an example, the well‐defined position of the surface exposed side chains in the presented structure facilitates its modification to incorporate known catalytic triads without altering the overall fibril properties. Also, it facilitates the design of small binding moieties to specifically interact with small molecules as heavy metals, pesticides, drugs, or toxins to develop highly specific filters and biosensors. Therefore, our structural, kinetic, and catalytic analyses, together with the available *CsgA* structures,^[^ ^16^, ^34^ ^]^ provide a foundation to exploit a rational design of functional bacterial‐amyloid‐based materials.

### 3.5 Note on Alternative *FapC* Structure

Two weeks after the publication of our brief description of our *FapC* structure in a review^[^ ^36^ ^]^ and a few days before we submitted the present paper, Hansen et al. posted a paper with a *FapC* UK4 structure on BioRxiv.^[^ ^46^ ^]^ Gratifyingly, our two structures are broadly similar. However, it would be remiss of us not to point out some issues with their structure. The authors claim a resolution of 3.3 Å, but this is likely to be closer to 4 Å as indicated by the lack of sidechain density in the core. Only the backbone of the inner core of cross‐β layer has visible electron density, and there is none for side chains even in the core. We also note the rather poor fitting of the AF3 predicted structure to the electron map. The main chain of the core is not well accommodated in the density map, and other parts of the cross‐β layer are fitted somewhat arbitrarily in isolated density map pots. The side chains of several residues in different IRs (Thr47/Tyr112/Glu175), which appear to be assigned to distinguish the three IRs, lack electron density for the main chains of these residues in the disordered region. Consequently, their assignment of the layers is different to our assignment. Just as the current *CsgA* structure is not of sufficient resolution to distinguish individual side chains, we do not believe that the proposed structure in ref. [46] is sufficiently robust to reveal the assembly mechanism of cross‐β stacking with IRs of FuA proteins. By contrast, our 3.2 Å electron density map allows us to fit the main chain of *FapC* very well, all the side chains of the inner and outer core are resolved and most side chain on the surface are also resolved, providing a robust model for the structure of this bacterial amyloid.

## 4 Experimental Section

### Bioinformatic Analysis

To create logo motifs for IRs in *FapB* and *FapC* genes, hmmsearch (HMMer v3.4) was applied to the GTDB database (v214) using Fap operon HMMs^[^ ^25^ ^]^ and an *e*‐value cutoff of 10^−5^, generating both *tblout* and *domtblout* output. *tblout* provided hits based on the summed bit scores of every imperfect repeat which the HMM detects within a gene, while *domtblout* provided every repeat detected by the HMMs in every gene, giving the locations of the repeats. These repeats were analyzed in all *FapB* and *FapC* genes located within a *Fap* operon, extracting the 3 repeats from each of these genes and designating them IR1–IR3 based on their order in the gene. The R package ggseqlogo was used to create sequence logos for repeats IR1, IR2, and IR3, as well as all of them combined. The analysis was elaborated upon in the results section. All code used to generate the results could be found at https://github.com/AOHD/Fap_analysis_public.

### Structural Prediction

Predictions of the structures of dimers of individual variants were performed using the AF3 web server (https://golgi.sandbox.google.com/) using at least 5 recycles of 10^9^ seeds (i.e., previously predicted structures used as reference for the next prediction within the same run). Additionally, 20 recycles were run in auto mode. Models were ranked using pLDDT, pTM, and ipTM scores. Due to limited number of FuA structures available for training, predicted conformations were considered reliable if at least one of the following criteria was fulfilled: a pLDDT score of at least 70 for the fibril core (disregarding disordered regions) or the combined pTM and ipTM scores were at least 0.5 and 0.6, respectively.

### Expression and Purification of *FapC* Constructs

Synthetic genes corresponding to UK4, PAO1, Pf5, and F1 wt *FapC* proteins were designed. These genes were then cloned into the pET22b vector and attached to a 6 × His tag at the end of the vector by Genscript (Nanjing, China) for subsequent purification. All *FapC* UK4 mutants were constructed using the *FapC* UK4 plasmid as a template. The plasmid was transformed into *E. coli* BL21 (DE3) cells and induced with 1 mm IPTG at OD_600_ ≈ 0.8. After 4 h at 37 °C, cells were pelleted at 5000 × *g* for 15 min, lysed with Buffer A (50 mm Tris/HCl, pH 7.4, 7.5 m guanidine hydrochloride, 20 mm imidazole), and stirred overnight at 4 °C. After centrifugation at 12 000 × *g* for 30 min at 4 °C, the supernatant was carefully collected. The Ni–NTA Beads 6FF gravity column was initially equilibrated with 5 column volumes (CV) of Buffer A. Subsequently, the supernatant was loaded onto the column, followed by washing with 5 CV of Buffer A. Finally, the *FapC* protein was eluted using Buffer B (50 mm Tris/HCl, pH 7.4, 7.5 m guanidine hydrochloride, 500 m imidazole), verified by SDS‐PAGE and stored at −80 °C. Before use, the protein was desalted into 50 mm Tris*/*HCl (pH 7.4) using a Zeba Desalter spin column (Thermo Fisher Scientific). The desalted protein was immediately placed on ice, and its concentration was determined by absorbance at 280 nm.

### ThT Fibrillation Assays of *FapC* Mutants

Fibrillation of *FapC* and mutants was monitored by ThT fluorescence in 96‐well transparent plates (Nunc, Thermo Fisher Scientific). 15 µm *FapC* (wt or mutant) was incubated with 50 mm Tris/HCl (pH = 7.4) and 40 µm ThT without shaking with continuous monitoring for 120 h. ThT fluorescence was monitored in an Omega microplate reader (BMG, Ortenberg, Germany) at 37 °C with excitation/emission at 448/485 nm and gain 1100. 100 µL of the mixed system was added to each well and fourfold technical replicates were carried out.

For seeding and cross‐seeding assays, 1–15 µm monomeric *FapC* UK4, PAO1, Pf5, and F1 were aggregated with 40 µm ThT. Mature fibrils formed at the highest concentration (15 µm) were then collected after the ThT kinetics reached a plateau. These mature fibrils were sonicated using a probe ultrasonicator (Qsonica) at 25% power and 30 s per cycle for 3 cycles. The resultant fibril seeds were mixed with 5 µm of *FapC* UK4 monomer using 2.5–10% seeds v/v, and 75 µL of the mixture was pipetted into a 96‐well plate. ThT fluorescence was monitored in a FLUOstar Omega microplate reader as described above.

### CD Secondary Structure Measurements

CD spectra were recorded using a Chirascan CD spectrophotometer (Applied Photophysics, Surrey, UK). Freshly prepared monomeric samples and fibrillated samples incubated at 37 °C for 72 h were adjusted to a final concentration of 0.48 mg mL^−1^ (20 µm) and placed in a 1 mm pathlength quartz cuvette. Measurements were conducted at 25 °C. The secondary structure composition was estimated using the DichroWeb online analysis tool.^[^ ^47^ ^]^

### FTIR Secondary Structure Measurements

FTIR spectroscopy analysis was performed using a Tensor 27 spectrometer (Bruker Optics Inc, Billerica, MA, USA). Approximately 3 µL of each sample was dried on the ATR crystal under dry nitrogen. Spectra were recorded in the range of 1800–1500 cm⁻¹ with a nominal resolution of 2 cm⁻¹ and 32 scans. Fourier self‐deconvolution and determination of band position of the original amide I band were achieved using PeakFit v4.12 software (SPSS Inc., Chicago, IL).

### AFM

*FapC* UK4, PAO1, Pf5, and F1 samples were prepared as follows. A 10 µL droplet of 0.5 mg mL^−1^ *FapC* solution was deposited to a freshly cleaved mica substrate and incubated for 5 min. The surface was subsequently rinsed with Milli‐Q water and gently dried under a stream of N₂ gas. AFM characterization was performed under ambient conditions in tapping mode and PeakForce quantitative nanomechanical mapping mode by Dimension system (Bruker, Billerica, MA, USA). Imaging was conducted with an RFESPA‐75 cantilever (Bruker, Billerica, MA, USA), which had a nominal spring constant of ≈3 nn nm^−1^, a resonance frequency of ≈75 kHz, and a tip radius of ≈10 nm. Images were acquired at scan rate of 1 Hz with a resolution of 512 × 512 pixels. To ensure reliable statistical analysis of *FapC* fibril formation, multiple regions of each sample were imaged. Image processing and data analysis were carried out using NanoScope Analysis 3.0 software (Bruker Billierica, MA, USA).

### SAXS

All SAXS data were obtained using the in‐house flux‐optimized Bruker AXS (Karlsruhe, Germany) SAXS NanoStar instrument with a Ga liquid metal jet source from Excillum (Kista, Sweden) and home‐built scatterless slits at Aarhus University.^[^ ^48^ ^]^ All samples were measured for 1800 s at 20 °C. Buffer and noise contributions were measured and subtracted, the intensity was normalized to absolute scale using scattering from water and finally logarithmically rebinned using the SUPERSAXS software package (J. S. Pedersen and C. L. P. Oliveira unpublished). Scattering intensity was given as a function of the scattering vector *q*, *q* = (4πsin(*θ*))/*λ*, where 2*θ* is the scattering angle and *λ* is the X‐ray radiation wavelength, *λ*_Ga_ = 1.34. The first step of the modeling was performed using geometrical models similar to the fibril model described before^[^ ^49^ ^]^ where a fibril was described by a cylindric fibril with an ellipsoid cross‐section with polymer contributions to account for disordered parts originating from loops and fluctuating regions. The cylinder had radius *R*, cross‐section axis ration *e*, length *L*, and a grading of the surface given by *σ*. The resulting scattering was described as the sum of the scattering from the components, the form factor of the cylinder *P*_cyl_ (*q*,*R*,e,*L,σ*) normalized to *P*_cyl_ (*q* = 0*,R*,e,*L,σ*) = 1 and the form factor of a Gaussian chains with radius of gyration *R*_g_, *P*_pol_(*q*,*R*_g_), also normalized, for the contribution from disordered regions. The intensity expression was

(1) $$I\left(q\right)=S_{\mathrm{cyl}}P_{\mathrm{cyl}}\left(q,R,\varepsilon ,L,\sigma \right)+S_{\mathrm{pol}}P_{\mathrm{pol}}\left(q,R_{g}\right)+b$$

where *S*_cyl_ and *S*_pol_ are scale factors and *b* is included for describing any residual background.

The overall length was fixed at *L* = 100 nm, which was so large that it did not influence the fit in the region of the SAXS data. Furthermore, the grading of the surface was fixed at *s* = 0.5 nm.

The next step of the modeling was based on AF3 predictions,^[^ ^37^ ^]^ since this included the whole *FapC* sequence, whereas the cryo‐EM structure lacked mobile regions such as the termini and linkers. Longer fibrils were constructed from the monomers of the variant using the script generated with help from OpenAI^[^ ^50^ ^]^ and Copilot in Microsoft 365.^[^ ^51^ ^]^ These fibrils were so long that the overall length did not influence the calculated scattering in the measured *q* range. For UK4, the AF model was compared with the experimentally determined structure from cryo‐EM, giving a RMS deviation of only 2.4 Å for the superposed structure. For the UK4, the N‐ and C‐terminal loops were moved manually to introduce some disorder. For the other variants, the AF structures were used without further modification. The scattering intensities of the models were calculated using AUSAXS (available at https://github.com/AUSAXS/). The program used the Debye equation to calculate the scattering, and a hydration layer was added to account for the higher electron density of the nearby water molecules. The program optimized an overall scale factor and a constant by minimizing reduced *χ* ^2^.

### Negative Stain Transmission Electro Microscopy

A 200‐mesh carbon‐coated copper grid was subjected to glow discharge treatment at 25 mA for 1 min. 2.6 µL of *FapC* fibrils were deposited onto the grid and incubated for 2 min, after which liquid was removed from the side of the grid with filter paper and 3.3 µL of 2% w/v uranium acetate was added to the stain for 1 min in two cycles. After blotting dry the dye solution, the copper mesh stand was air dried for 10 min and imaged on a Talos L120C G2 transmission electron microscope (Thermo Fisher Scientific).

### Cryo‐EM Data Collection and Processing

To ensure the uniform dispersion of the fibril samples during imaging, 10 mm Tris (2‐carboxyethyl) phosphate hydrochloride was added to all the fibrils for cryo‐EM data collection during the incubation process. Sample solutions were applied to glow‐discharged Quantifoil R1.2/1.3 200‐mesh Au grids (2 min, 25 mA) and plunge‐frozen in liquid ethane using a Vitrobot Mark IV (FEI) with a sample volume of 2.6 µL per grid. cryo‐EM data were acquired on a Titan Krios transmission electron microscope (Thermo Fisher Scientific) operated at 300 kV, with a nominal physical pixel size of 0.932 Å px^−1^ and a total accumulated dose of 40 e^−^ Å^−2^ per image. Data processing followed the workflow in Figure S1 (Supporting Information). From 2006 collected microscopy images of the *Pseudomonas* sp. UK4, *FapC* fibril sample, motion correction, and contrast transfer function (CTF) estimation were performed using MotionCor2^[^ ^52^ ^]^ and CTFFIND‐4.1.827,^[^ ^53^ ^]^ respectively, within RELION 4.0.^[^ ^54^ ^]^ Particles were autopicked with Topaz v0.2.5^[^ ^55^ ^]^ and extracted at box sizes of 720 and 360 pixels, maintaining an interparticle distance of 10% of the box size. 2D classification of 720 pixel box size particles resolved two populations: twisted fibrils (26.7% of particles) and nontwisted fibrils of variable widths (58.7% of particles), with the remaining 14.6% classified as unstructured junk particles. Particles from the twisted subset (360 pixel box size) were selected for initial 3D reconstruction. Helical symmetry parameters were determined via 2D class inspection, refined to a rise of 4.8 Å and twist of −2.0° (assuming homogeneous layer stacking). Two rounds of 3D classification (3 classes per round) isolated high‐quality particles for gold‐standard refinement, yielding initial near‐atomic resolution maps. Following CTF refinement and Bayesian polishing, a final map with 4.8 Å helical rise was generated. Evidence of heterogeneous layer stacking during model building prompted another refinement using a three‐layer repeat unit (14.4 Å rise, −6.0° twist), which resolved structural ambiguities and supported atomic model building.

### Atomic Model Building

The cryo‐EM map of *FapC* fibrils exhibited a main‐chain conformation nearly identical to that predicted by AF3,^[^ ^56^ ^]^ enabling its use as the initial model. Rigid body fitting of the AF3 model into the 4.8 Å helical rise map revealed strong agreement with the density, requiring only minor adjustments in Coot.^[^ ^57^ ^]^ The AF3 model comprised three layers, each corresponding to one of three *FapC* repeats. To evaluate whether the fibril structure arose from homogeneous or heterogeneous layer stacking, side‐chain densities were meticulously analyzed. None of the three repeats alone fully matched the cryo‐EM map, indicating structural heterogeneity. Specifically, side‐chain densities near Gly65 (repeat 1), Leu130 (repeat 2), or Gly203 (repeat 3) suggested that repeat 2 contributed to the fibril assembly. Additionally, extra densities on the central slice of the 3D reconstruction aligned with a flexible loop unique to repeat 3, further supporting multilayer stacking. Consequently, the cryo‐EM data were reprocessed using a 14.4 Å helical rise (three‐layer repeat symmetry). The refined map validated this model: 1) one layer displayed side‐chain densities matching Leu130 and Gln150 (repeat 2); 2) another layer exhibited a histidine‐shaped density at His182 (repeat 3), stabilized by hydrogen bonding to the main chain, unlike lysines at equivalent positions in repeats 1 and 2, which lacked stabilizing interactions; 3) the remaining layer, lacking densities at Leu130 (repeat 2) or His182 (repeat 3), was assigned to repeat 1. The final model, comprising three subunits (each with three layers) was refined with phenix.real_space_refine^[^ ^58^ ^]^ and validated using MolProbity^[^ ^59^ ^]^ within the PHENIX v.1.20.1‐4887 software package for several rounds until an acceptable Ramachandran plot, Molprobity score, and Clashscore values were obtained, which were deemed to be compatible with amyloid fibril structures featuring tight inter‐ and intralayer packings.

### Fibril Stability toward FA

The *FapC* wt and mutants were purified in 7.5 m GdmCl as described above. Immediately prior to aggregation, *FapC* samples were desalted on a PD25 column, protein concentration was determined through absorption at 280 nm, and the proteins were fibrillated as described above. The mature fibrils were centrifuged at 15 000 rpm for 30 min, after which the pellet was resuspended in 50 µL of pure water. 5 µL of resuspended fibrils were incubated at room temperature in 50 µL of different concentrations (0–100%) of FA for 30 min, then centrifuged at 15 000 rpm for 30 min. 20 µL of the supernatant was transferred into a new 1.5 mL EP tube and freeze‐dried, then dissolved in 20 µL of pure water and run on a SDS‐PAGE gel. The gel was scanned and imaged using a gel imager (ChemiDoc MP, bio‐rad, USA). The protein bands were quantitatively analyzed using ImageJ software and normalized to the band where the fibrils were dissolved in 100% FA. The normalized FA dissolution curve was plotted using GraphPadPrism 8 software.

### Catalytic Hydrolysis of *p‐nitrophenyl acetate (p*NPA) and *Para*‐Nitrophenyl Butyrate (pNPB)

*FapC* esterase and lipase activity were evaluated as described^[^ ^7^ ^]^ through the absorbance at 410 nm of the product *p*NP measured over 3 h. *p*NPA and *p*NPB solutions were prepared in 100% acetonitrile and stored up to 1 week at –20 °C. Prior to use, 10× stocks of the substrates were prepared in the reaction buffer (Tris/HCl 50 mm pH 7.4 supplemented with 40% acetonitrile for *p*NPA and concentrations below 30 mm of *p*NPB or 50–75% acetonitrile for stocks of 40 and 60 mm, respectively). Preformed *FapC* fibrils were prepared by incubation of 40 µm of *FapC* for 72 h at 37 °C in Tris/HCl 50 mm pH 7.4 and kept frozen at −20 °C until use. The reaction was performed in a 96‐well plate in presence or absence of 10 µm of preformed *FapC* fibrils diluted in Tris/HCl 50 mm pH 7.4. To the fibril mixture was added 15 µL of the 10× stock to a final volume of 150 µL, after which the plate was sealed and placed in a CLARIOstar platereader (BMG LABTECH, Ortenberg, Germany) previously incubated at 37 °C. Hydrolysis of *p*NPA and *p*NPB was followed by measuring the absorbance increase at 410 nm every 2–3 min for 3 h with double orbital shaking between measurements to avoid fibril precipitation. Initial production rates (*v*_0_) were calculated as the slope of the *p*NP production at the first 60 min (the first 10 min were not analyzed due to high noise and temperature instability). Each reported result was an average of at least three measurements. Each experiment included also blank samples of substrate diluted with buffer instead of *FapC* fibrils to follow substrate self‐degradation, which was subtracted from the initial rate calculations. All presented results included subtraction of buffer control.

### *p*NPP Esterolysis Kinetics

Production of *p*NP from *p*NPP embedded in unilamellar vesicles was followed for 10 h. The substrates were prepared as previously reported.^[^ ^8^ ^]^ Briefly, vesicles made of POPC/DOPC and *p*NPP (molar ratio 1:2) were prepared by dissolving the lipid components in chloroform/ethanol (1:1, v/v), left to dry, and stored at −20 °C until use. Vesicle solutions were resuspended in 50 mm Tris/HCl pH 7.4 and sonicated at room temperature for 15 min at 25% amplitude; the solution was incubated for 1 h at room temperature before use. Different concentrations of DOPC/POPC–*p*NPP vesicles (1 and 2 mm) were transferred to a 96‐well plate in the presence or absence of 10 µm of *FapC* fibrils. The plate was then sealed and placed in a CLARIOstar platereader (BMG LABTECH, Ortenberg, Germany) previously incubated at 37 °C, and the formation of *p*NP followed as described above for 10 h with background subtraction as above.

### *FapC* Fibril Recyclability

*FapC* fibrils were prepared in a 96‐well plate to a final concentration of 10 µm of 50 mm Tris/HCl pH 7.4, with a total volume of 135 µL, to which was added 7.5 µL of 16 mm *p*NPA (Tris/HCl 50 mm pH 7.4, supplemented with 40% acetonitrile). The plate was immediately sealed and placed in a CLARIOstar platereader (BMG LABTECH, Ortenberg, Germany) previously incubated at 37 °C to follow *p*NPA degradation as above described. After 5 h, another 7.5 µL of 16 mm *p*NPA (buffer as above) was added and the process repeated.

### Impact of Denaturing Conditions on *FapC* Catalytic Behavior

The resistance of *FapC* fibrils to harsh conditions was evaluated by measuring ThT fluorescence and the maintenance of the catalytic capacity after different kinds of treatment. 40 µm of *FapC* fibrils was incubated for 10 min either at 95 °C, in 3 m NaOH or in 20% FA. After thermal incubation, the fibrils were left to recover for 10 min to reach room temperature. Fibrils incubated in NaOH or FA were pelleted (13 500 rpm, 30 min) and washed with Tris/HCl 50 mm pH 7.4, followed by supernatant removal; the pH was evaluated in the supernatant after each round of centrifugation to ensure that neutral pH was reached. To assess resistance to physical stress, untreated fibrils were sonicated (3 cycles of 30 s ON/OFF at 25% amplitude) and kept on ice until use. The resultant treated fibrils were diluted in Tris/HCl 50 mm pH 7.4 in a 96‐wells plate to a final concentration of 10 µm (assuming no loss during pelleting and washing). Esterase capacity was then evaluated as described above. ThT analysis was performed by diluting treated and untreated *FapC* fibrils in Tris/HCl 50 mm pH 7.4 supplemented with 40 µm of ThT. The fluorescent emission was measured in a Cary Fluorimeter (Agilent, CA, USA) by exciting at 445 nm and measuring emission from 460 to 600 nm. Thermal stability was performed by exciting at 445 nm and collecting at 482 nm every 1 min, with a temperature increase/decrease of 1 °C min^−1^ with constant stirring. Finally, the effect of ionic strength on catalytic activity was assessed by following the esterase capacity as previously described in the presence of different concentrations of 0–2 m NaCl.

### Membrane Functionalization with *FapC* Fibrils

Surface functionalization was performed on PVDF and NC membranes. The membranes were manually prepared to fit on the wells, with a diameter of ≈7 mm. In the case of the PVDF, the membranes were activated with 30% ethanol and washed with milli‐Q water prior to use. Different amounts of *FapC* fibrils (0, 50, 100, and 200 µg) were added to the surface of the membranes and incubated at 45 °C until dry. The membranes were then placed in a 96‐well plate, and 135 µL of Tris/HCl 50 mm pH 7.4 added, followed by 15 µL of 10 mm of *p*NPA (Tris/HCl 50 mm pH 7.4 with 40% acetonitrile). The plate was immediately sealed and placed in a CLARIOstar platereader (BMG LABTECH, Ortenberg, Germany) preincubated at 37 °C. After 20 min of incubation with double orbital shaking, the supernatant was recovered, and absorbance was measured at 410 nm. Subsequently, the membranes were washed twice with 200 µL of Tris/HCl 50 mm pH 7.4. Finally, the catalytic capacity was tested as before using Tris and *p*NPA.

### Cytotoxicity Assays

Cytotoxicity of *FapC* fibrils was tested using ROS toxicity cellular assays (Abcam, Cambridge, UK) as described.^[^ ^60^ ^]^ Briefly, HEK293 cells were exposed to the cell‐permeable reagent 2′, 7′‐dichlorofluorescein diacetate which is converted to the highly fluorescent dichlorofluorescein upon oxidation. Cells were incubated with 100 µg mL^−1^ of other sonicated or nonsonicated *FapC* UK4 fibrils formed as described above and incubated for 1 h before measuring fluorescence (no significant changes were observed over longer periods). As positive controls, the oxidant *tert*‐butyl hydroperoxide and hydrogen peroxide were used. For comparison, cells were also exposed to 100 µg mL^−1^ of either fibrils or oligomers of α‐synuclein prepared as described.^[^ ^60^ ^]^

### Data Availability

The cryo‐EM map and atomic model of *FapC* fibrils from *Pseudomonas* sp.UK4 were deposited into the Worldwide Protein Data Bank and the Electron Microscopy Data Band with accession codes PDB 9U4U and EMD‐63855. All other relevant data were available from the corresponding authors upon request.

## Conflict of Interest

The authors declare no conflict of interest.

## Supporting information



Supporting Information

## Data Availability

The data that support the findings of this study are available from the corresponding author upon reasonable request.
