# Supplementary material for: Natural Design of a Stabilized Cross‐β Fold: Structure of the FuA FapC from Pseudomonas Sp. UK4 Reveals a Critical Role for Stacking of Imperfect Repeats
Source: Adv Mater. 2025 Jun 11;37(34):2505503. doi: 10.1002/adma.202505503 (PMC12392863; doi:10.1002/adma.202505503)
Supplement: Supplementary file 1 — Supporting Information [file ADMA-37-2505503-s001.pdf]

ADVANCED MATERIALS

Supporting Information

for *Adv. Mater.*, DOI 10.1002/adma.202505503

Natural Design of a Stabilized Cross-β Fold: Structure of the FuA *FapC* from *Pseudomonas* Sp. UK4 Reveals a Critical Role for Stacking of Imperfect Repeats

Yanting Jiang, Samuel Peña-Díaz, Zhefei Zhang, Anders Ogechi Hostrup Daugberg, Marcos López Hernández, Janni Nielsen, Qiaojie Huang, Shenghan Qin, Morten K. D. Dueholm, Mingdong Dong, Jan Skov Pedersen, Qin Cao*, Daniel E. Otzen* and Huabing Wang**

Natural design of a stabilized cross-β fold: Structure of the FuA FapC from *Pseudomonas* sp. UK4 reveals a critical role for stacking of imperfect repeats

Yanting Jiang, Samuel Peña-Díaz, Zhefei Zhang, Anders Ogechi Hostrup Daugberg, Marcos López Hernández, Janni Nielsen, Qiaojie Huang, Shenghan Qin, Morten K. D. Dueholm, Mingdong Dong, Jan Skov Pedersen, Qin Cao, Daniel E. Otzen and Huabing Wang

Supplementary Information

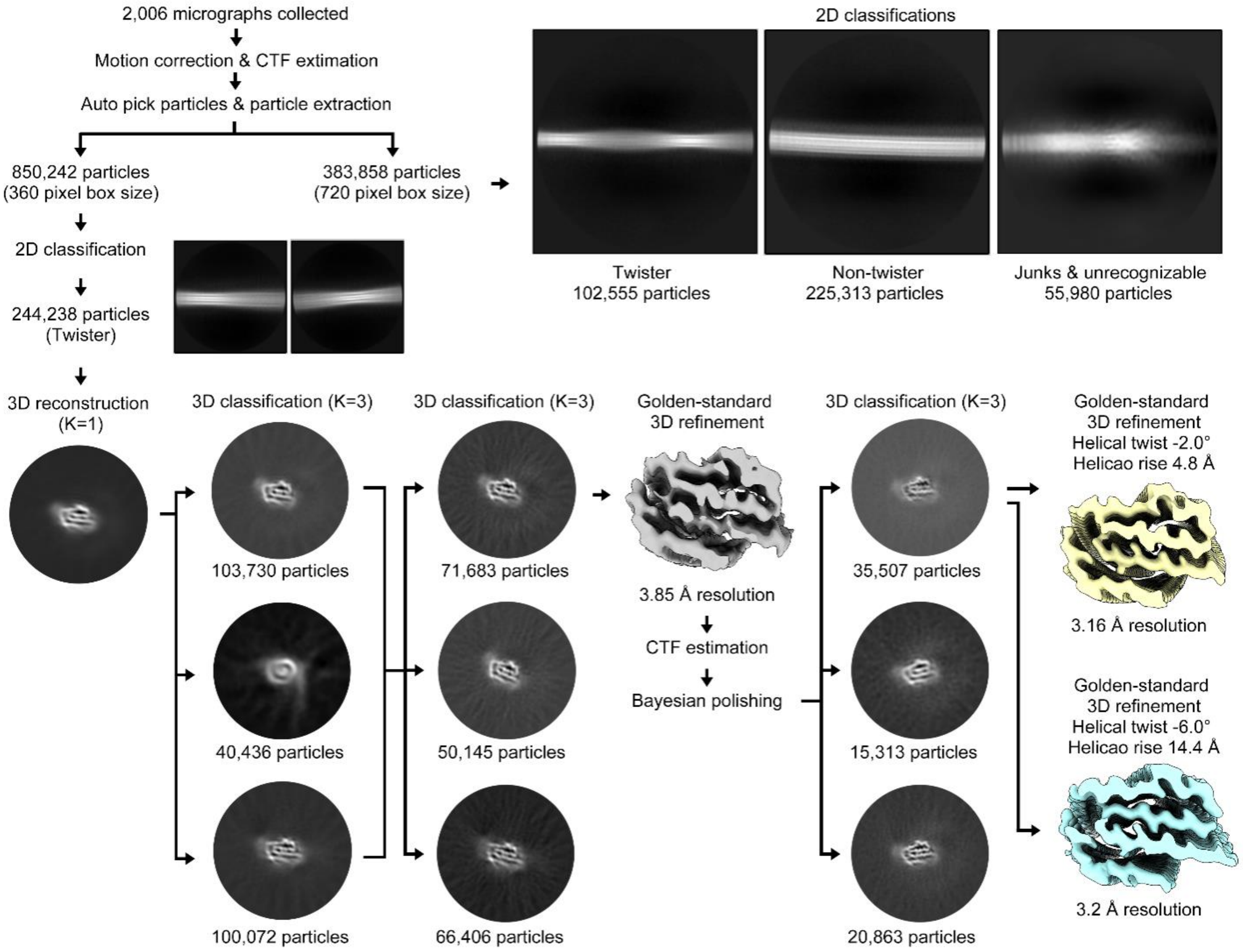

Figure S1. Data processing workflow of cryo-EM analysis of FapC fibrils.

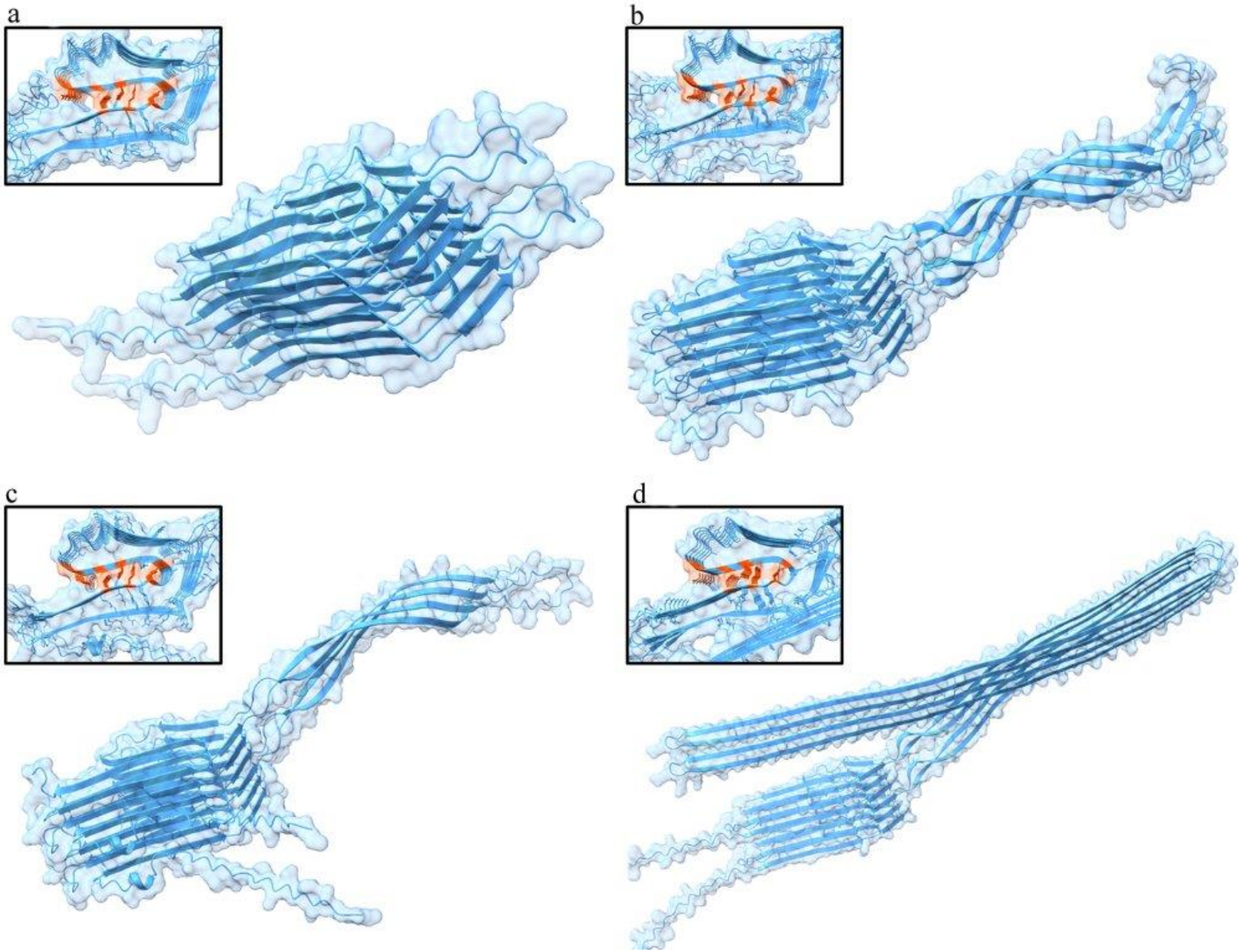

Figure S2. AF3 prediction of different FapC fibrils. Structural prediction of different FapC variants including UK4 (a), PAO1 (b), Pf5 (c) and F1 (d). Insets show the core of the amyloid fibrils depicting in red the conserved residues and in dark grey the hydrogen bonds.

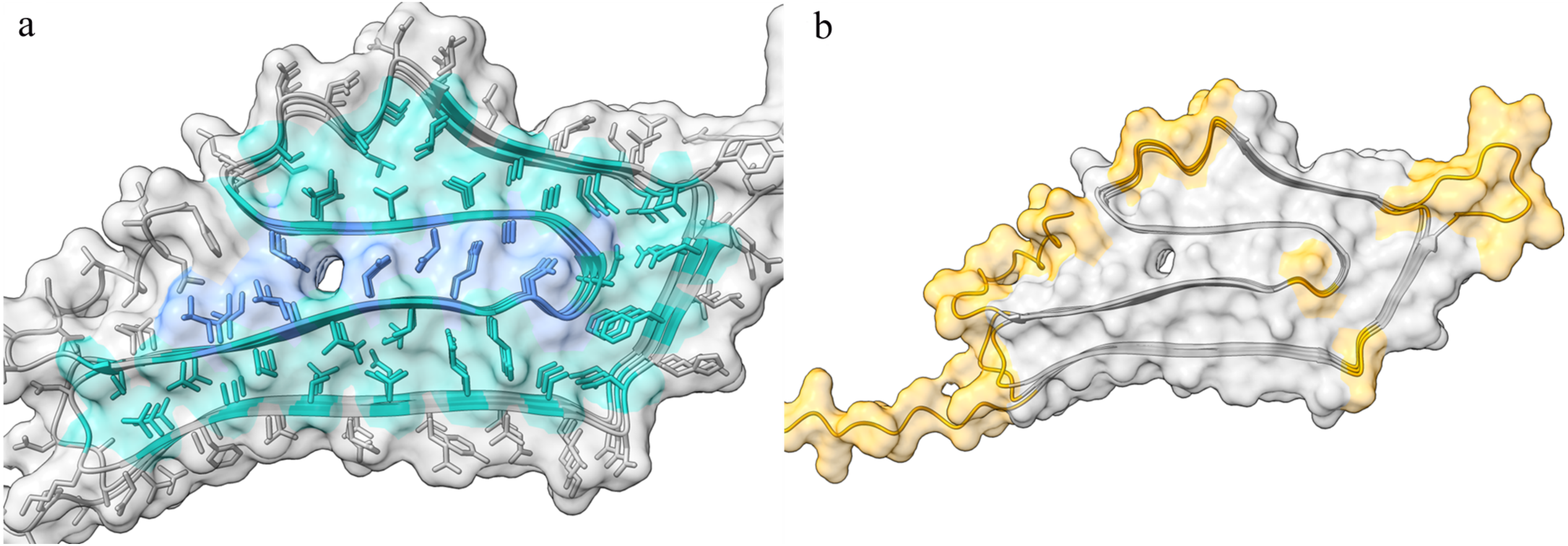

Figure S3. UK4 structure prediction. (a) Characterization of the inner cavity (blue), outer cavity (turquoise) and outer residues (grey). (b) Distribution of disordered regions (orange) and β-sheet forming residues (grey).

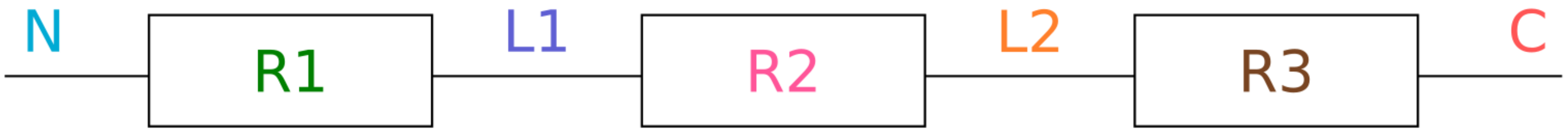

	N	R1	L1	R2	L2	R3	C
UK4:	59	38	27	39	35	38	14
Pf5:	77	39	28	39	103	38	5
PAO1:	64	39	30	37	119	38	13
F1:	74	38	34	38	259	37	3

Figure S4. Lengths of C-terminal, repeats and linker regions in the FapC genes of *Pseudomonas* species UK4, PAO1, Pf5 and F1.

a

\>PAO1
MGNEGGWHPPKPNPQSNNKGGATALVVDTQQNYNNKVSNFGTLNNASVSG
SIKDASGNVGVNVAAGDNNQQANAAALASADASFVFGTATASTSVLQSGY
GNTLNNYSNPNTASLSNSANNVSGNLGVNVAAGNFNQQKNDLAAAVSNGQ
YSTAGSAASQTSTGNTTVNSANYAYGGTYVSLKLNADGSYKGTSDQIGDV
YLDTWEGQTHPGGSNTGHIDVDSQAQGAKDLNHDGGAFAFKEKGDVDLKG
TVSGFIPAIVGFKTPVTNNASLSNSLQNVSGNVGVNIAAGGGNQQSNSLS
IAAGCSSCPAGGESLGF

\>Pf5
MGGNDHGNNGGHGNGHGDHDQGPDLSTLLQITAGAGAAVIDVQNSNTNVV
KNQGTNNNAKADNSLNNSNGNMGANVAAGDGNQQDNAAALATADESFIFG
SAVAASSATQVNNNNYVKNSSTQNNAVLNNSGNNGSGNIGINVTAGDFNQ
QKNNLAIAVSGGRVATAAASANQQSTNLVVDNKGVQAYKKDTLSGGFVAS
GTYKGTGSGKIEGGDDHSGHGYGDNKGHGNDDQKFTFKEQGTIELAGYWT
QQVLTKDGWKNPVVNNASMTNSMNGFSGNGGANVSAGVGNQQSNSLSIAA
GCKACM

\>F1
MGQNDRRNDHHNGHHNNGHHTPPPTKIPVYATANAWDTQTSTRNRISNEG
TINEAEMSDSGTGSSGNVGINVAAGNGNQQDNAAAIANAGSDSSLDNSFV
FGMASATADVVQTSRNNRVDNYSTQSSAVMSGSASGAEGNIGINVAGGDL
NQQKNTMAIANTGAPLGNATATASANQDGPGLIVNNSADRTYRVDTITIT
KSASGSSSKEGSFNSTDDRSSSSSFSVAGAQSASSSGSSGWNASGSQSSN
ASGSHNSSASGSFGWDASGSASGSHSASSSASLAASLDAAANATRTFTTD
DGRRERTRSSTFDGSLSASLDVSVDKSHESAYDSSFEKAFDSNYDKAHQS
SYDKSKESSYTKSHDSSYENASASEFSKSGEQSKQSSNDVSKSYSESSAY
DLSNTVSFQVLTPTGWANPVTNTATLSGSVNGGSGNLGVNVAAGVGNQQS
NSLAISNTSF

b

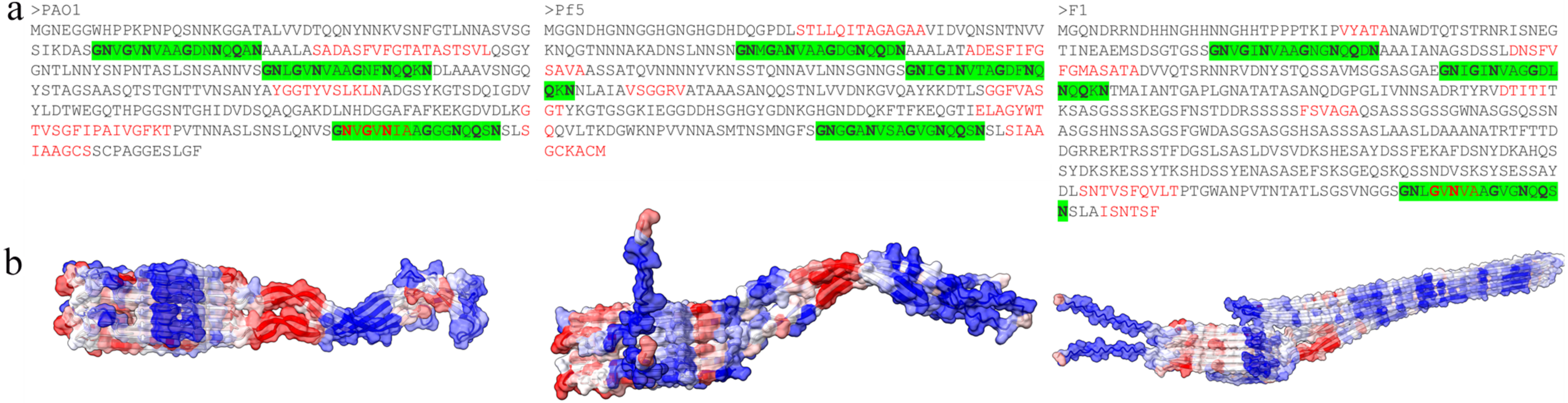

Figure S5. Aggregation propensity of PAO1, Pf5 and F1 variant. (a) Linear prediction of APRs using Aggrescan. Residues with aggregation propensity are shown in red; repeats and highly conserved residues are shown in green and bold. (b) Structural prediction of APRs using Aggrescan4D. Low aggregation propensity is shown in blue, while high aggregation tendency is shown in red.

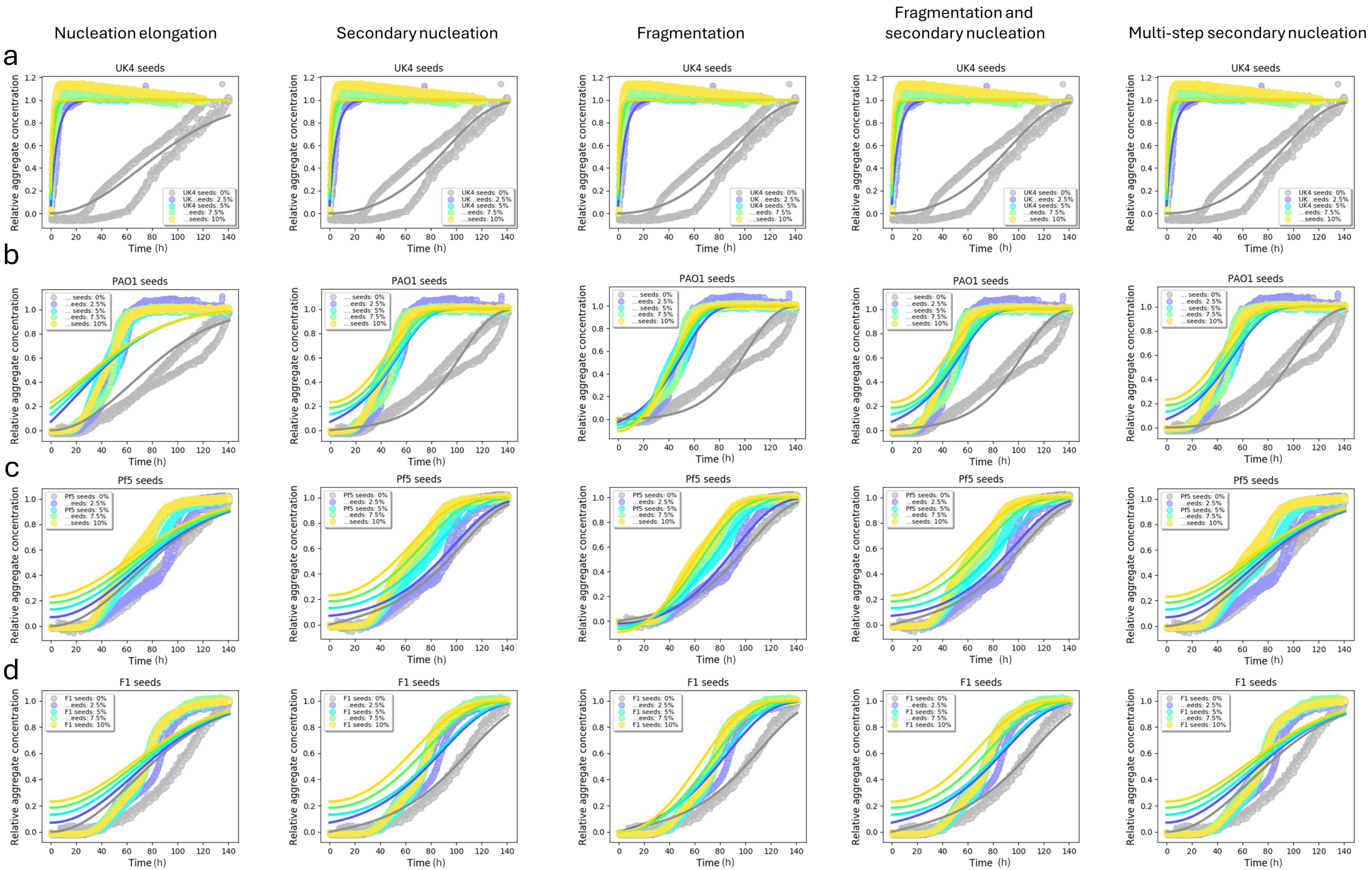

Figure S6. AmyloFit comparative evaluation of the aggregation of monomeric UK4 FapC in presence of seeds made from (a) UK4, (b) PAO1, (c) Pf5 and (d) F1 FapC. Time units are in hours.

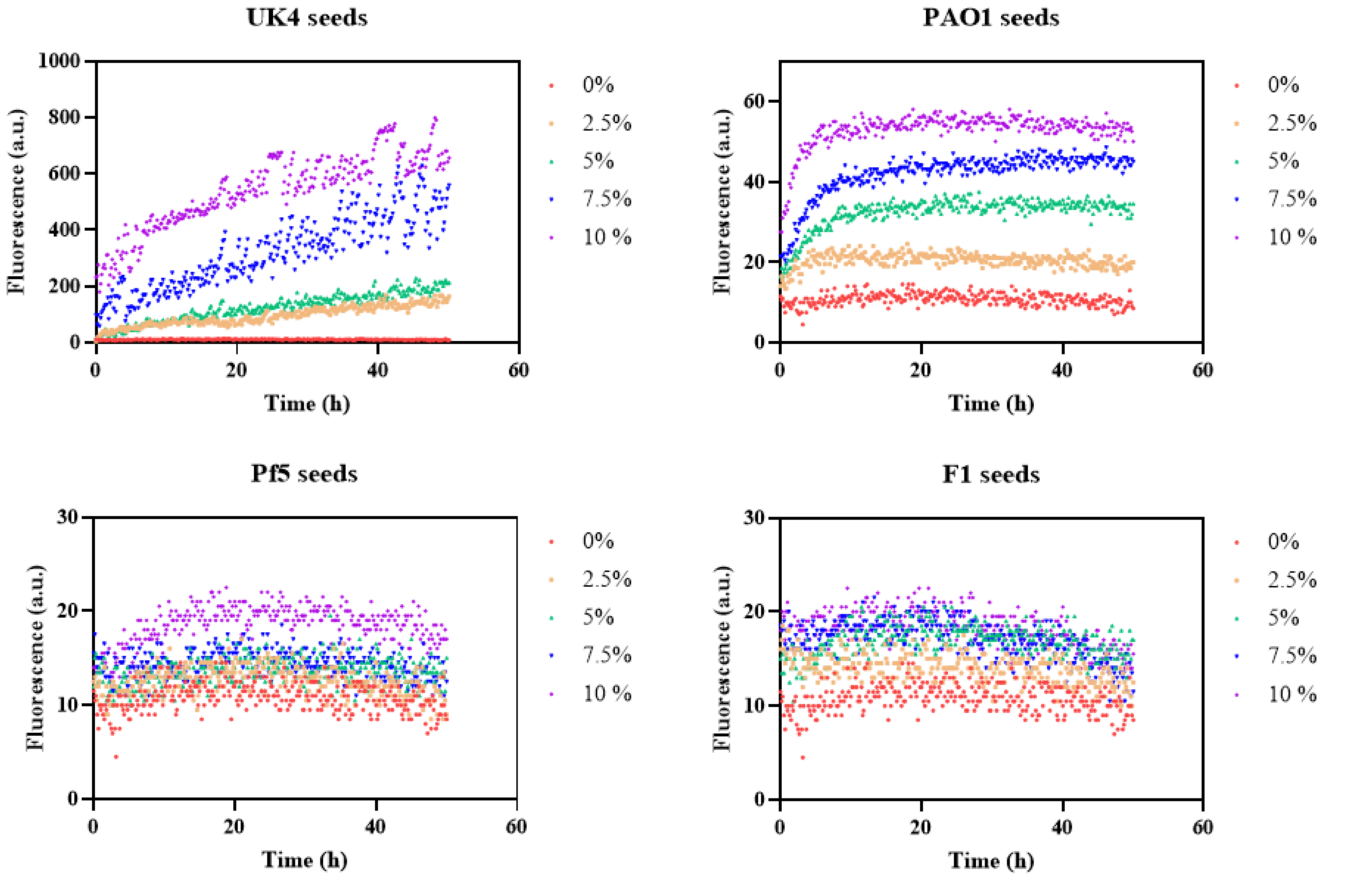

Figure S7. Fibrillation of monomers of PAO1 FapC alone (0%) and in the presence of 2.5-10% (w/w) seeds made with different FapC strains.

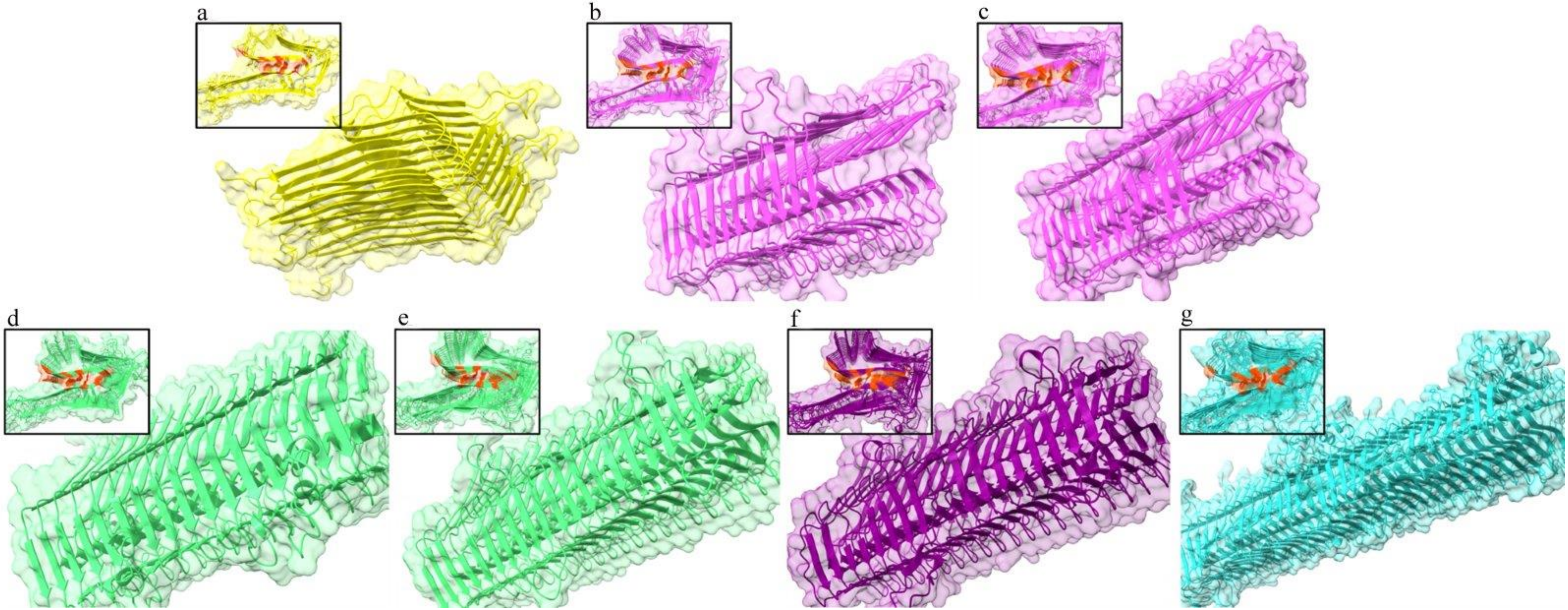

Figure S8. AF3 prediction of different FapC fibrils. Structural prediction of different fapC variants including (a) NZ_QJRX01000007.1_45 (*Pseudomonas E alcaligenes B*), (b) NZ_JALQCS010000001.1_182 (UBA2486 sp023277115), (c) NZ_WNJL01000037.1_125 (*Acidithiobacillus ferrianus*), (d) NZ_JACKZA010000010.1_131 (*Acidithiobacillus sp015100155*), (e) JAJYRD010000073.1_41 (*Acidiferrobacter sp021794275*), (f) JAKBUS010000016.1_3 (*Acidiferrobacter sp021841585*) and (g) NZ_AEVS01000081.1_90 (*Vibrio brasiliensis*), which differs in the linker size and number of repeats: 4 repeats (yellow), 6 repeats (pink), 8 repeats (green), 10 repeats (violet) and 16 repeat (turquoise). Insets show the core of the amyloid fibrils depicting in red conserved residues and in dark grey hydrogen bonds.

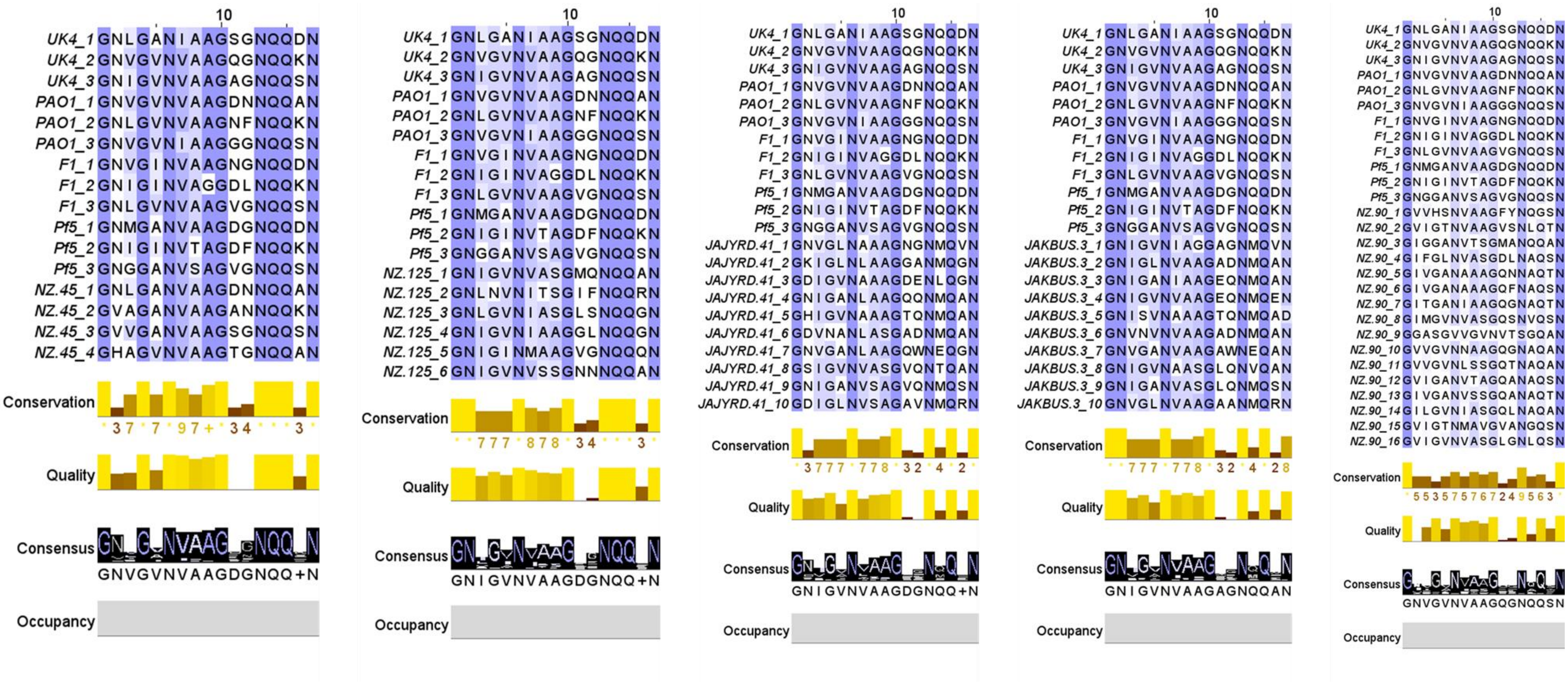

Figure S9. Conservation degree of larger repeat variants. Representative comparison of the conservation degree of NZ_QJRX01000007.1_45, NZ_WNJL01000037.1_125, JAJYRD010000073.1_41, JAKBUS010000016.1_3 and NZ_AEVS01000081.1_90. Conservation levels are shown below and highlighted using the Clustal and Jalview (shown in yellow) conservation groups. Individual residues are color-shaded based on conservation levels using Blosum62 matrix (30% thresshold).

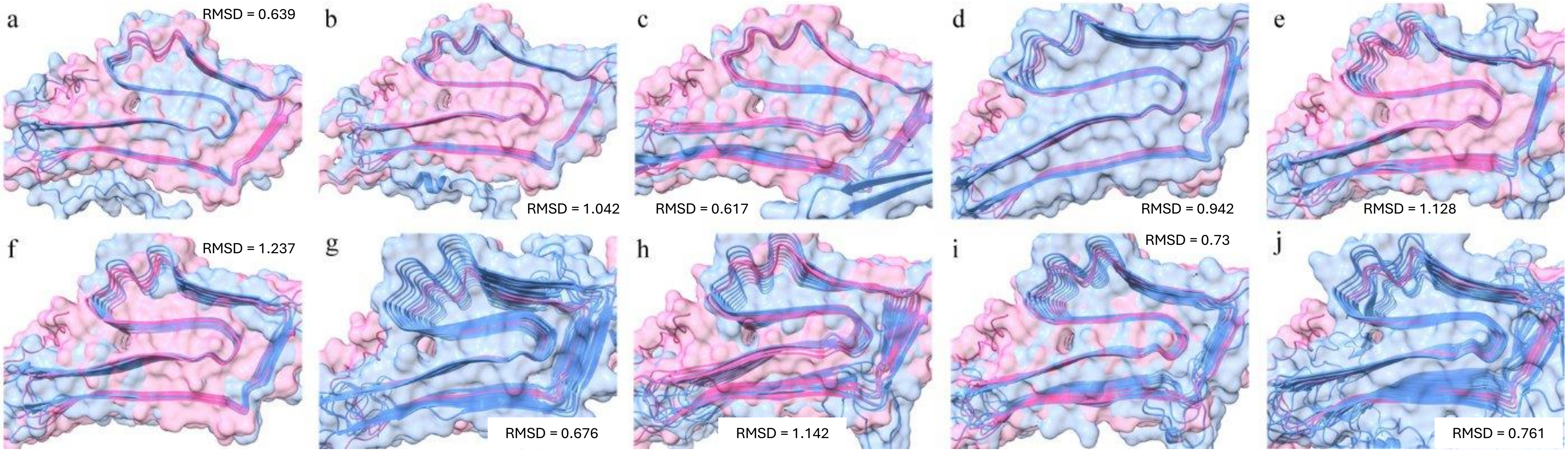

Figure S10. Structural homology within the FapC family. Representative images of FapC UK4 (pink) compared to other FapC variants in blue: (a) PAO1 *(Pseudomonas aeruginosa*), (b) Pf5 (*P. fluorescens Pf5),* (c) F1 (*P. putida F1*), (d) NZ_QJRX01000007.1_45, (e) NZ_JALQCS010000001.1_182, (f) NZ_WNJL01000037.1_125, (g) NZ_JACKZA010000010.1_131, (h) JAJYRD010000073.1_41, (i) JAKBUS010000016.1_3, and (j) NZ_AEVS01000081.1_90 (organisms e-j listed in legend to Fig. 4). RMSD scores are shown in Å.

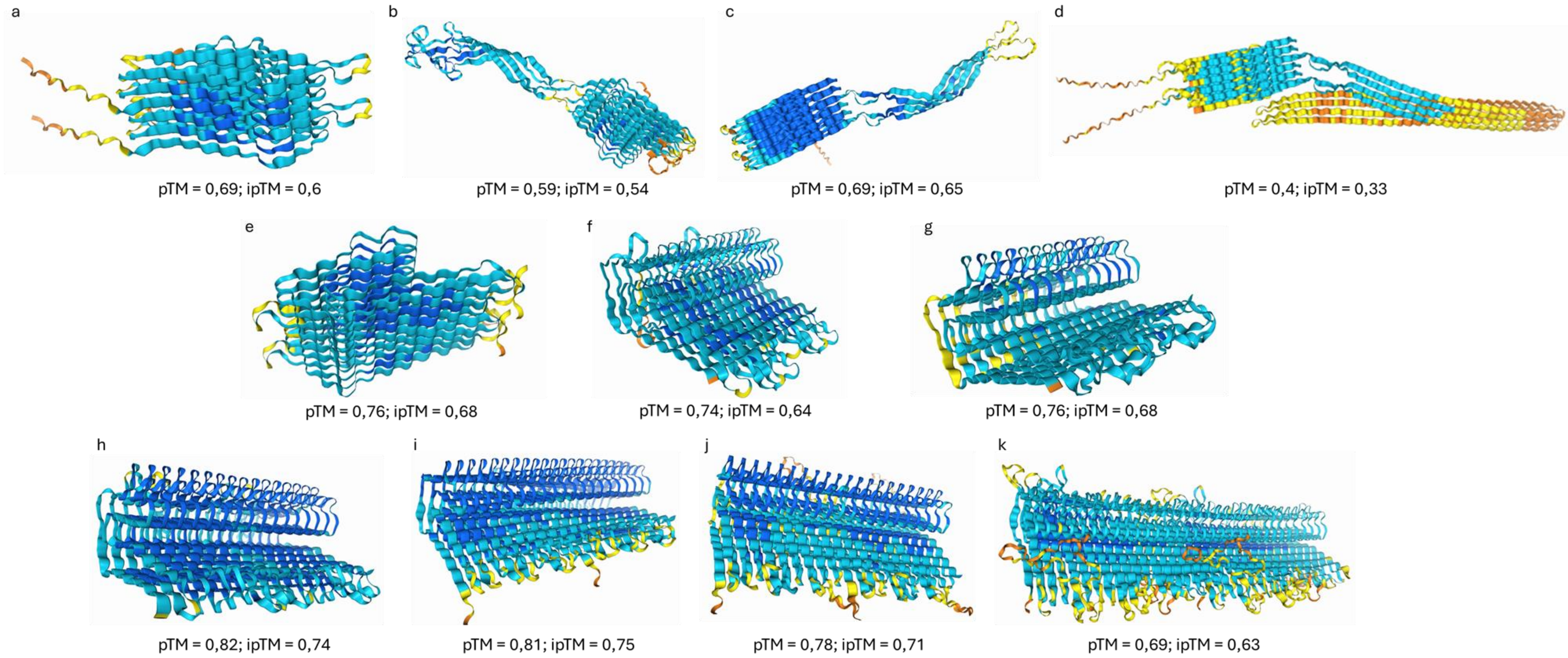

Figure S11. AF3 prediction of different FapC fibrils. Scores of the structural prediction of different FapC variants: (a) UK4, (b) PAO1, (c) Pf5, (d) F1, (e) NZ_QJRX01000007.1_45, (f) NZ_JALQCS010000001.1_182, (g) NZ_WNJL01000037.1_125, (h) NZ_JACKZA010000010.1_131, (i) JAJYRD010000073.1_41, (j) JAKBUS010000016.1_3 and (k) NZ_AEVS01000081.1_90. Colors indicate plDDT values with dark blue indicating plDDT >90; light blue, 70-90; yellow, 50-70; orange, <50.

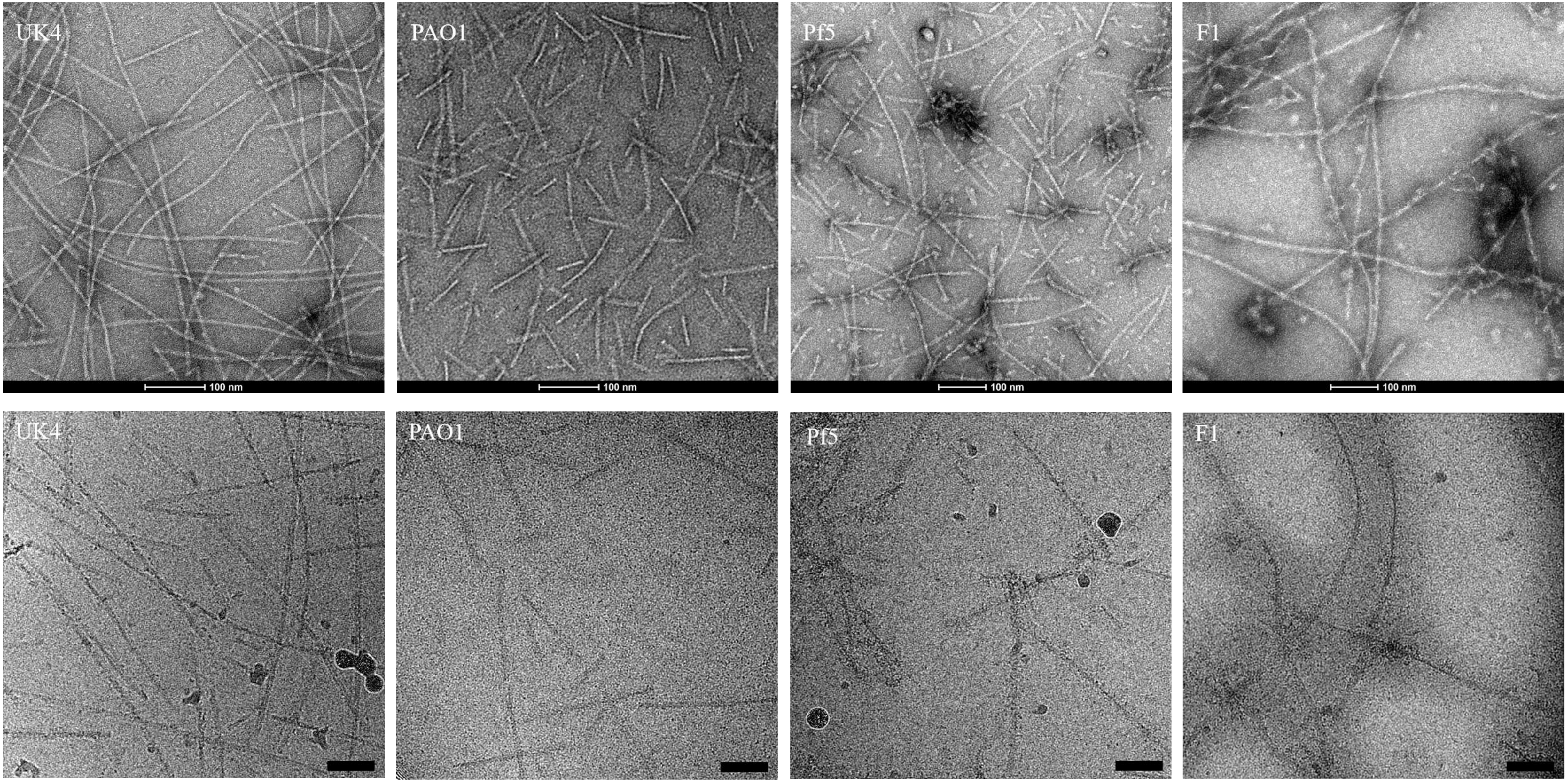

Figure S12. (Top) EM negative stain and (Bottom) cryo-EM images of UK4, PAO1, Pf5, F1 fibrils (300kv). Scale bar = 50 nm

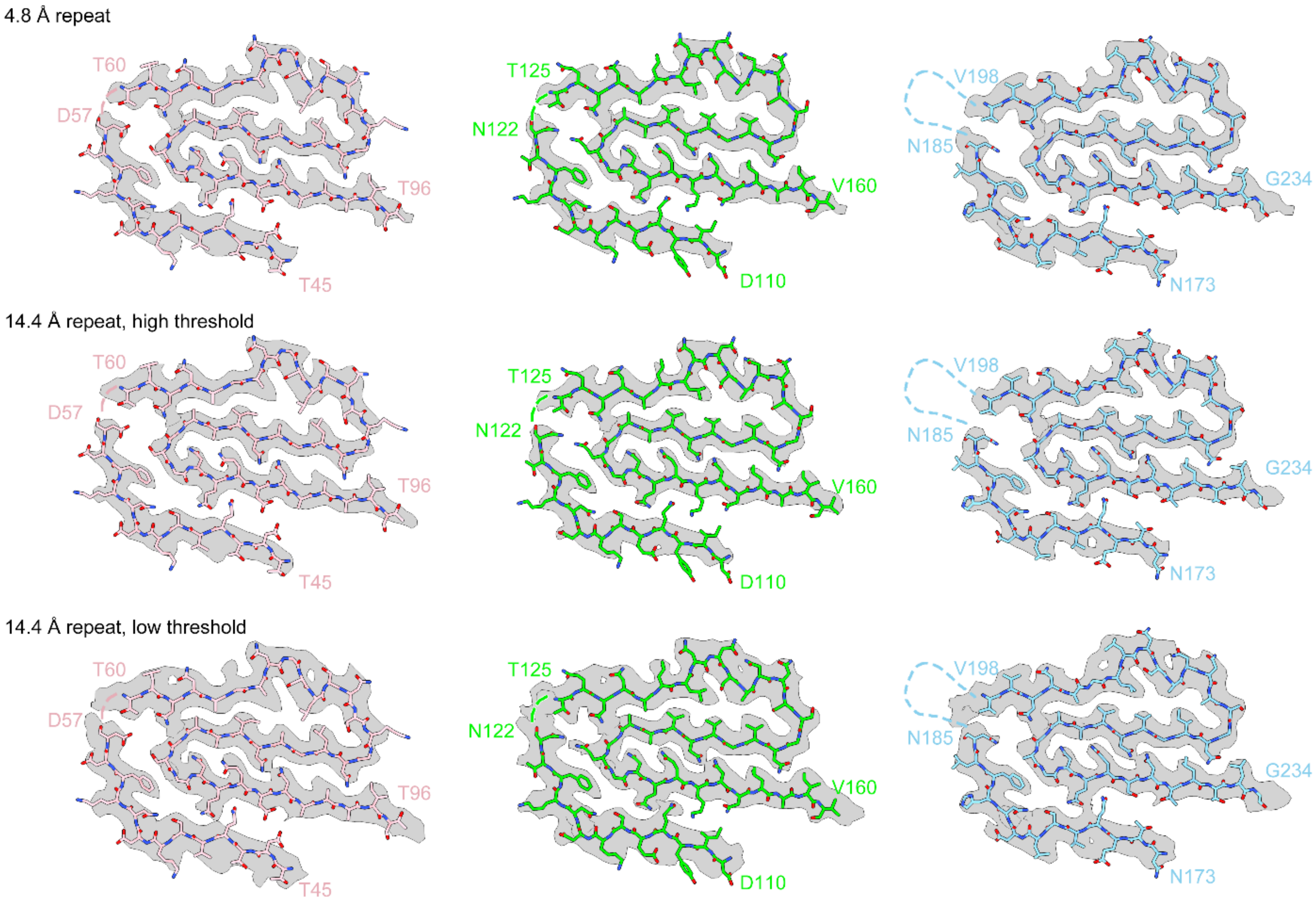

Figure S13. Fitting the model of three imperfect repeats to the reconstructed 3D map (gray) using different repeat units and different thresholds.

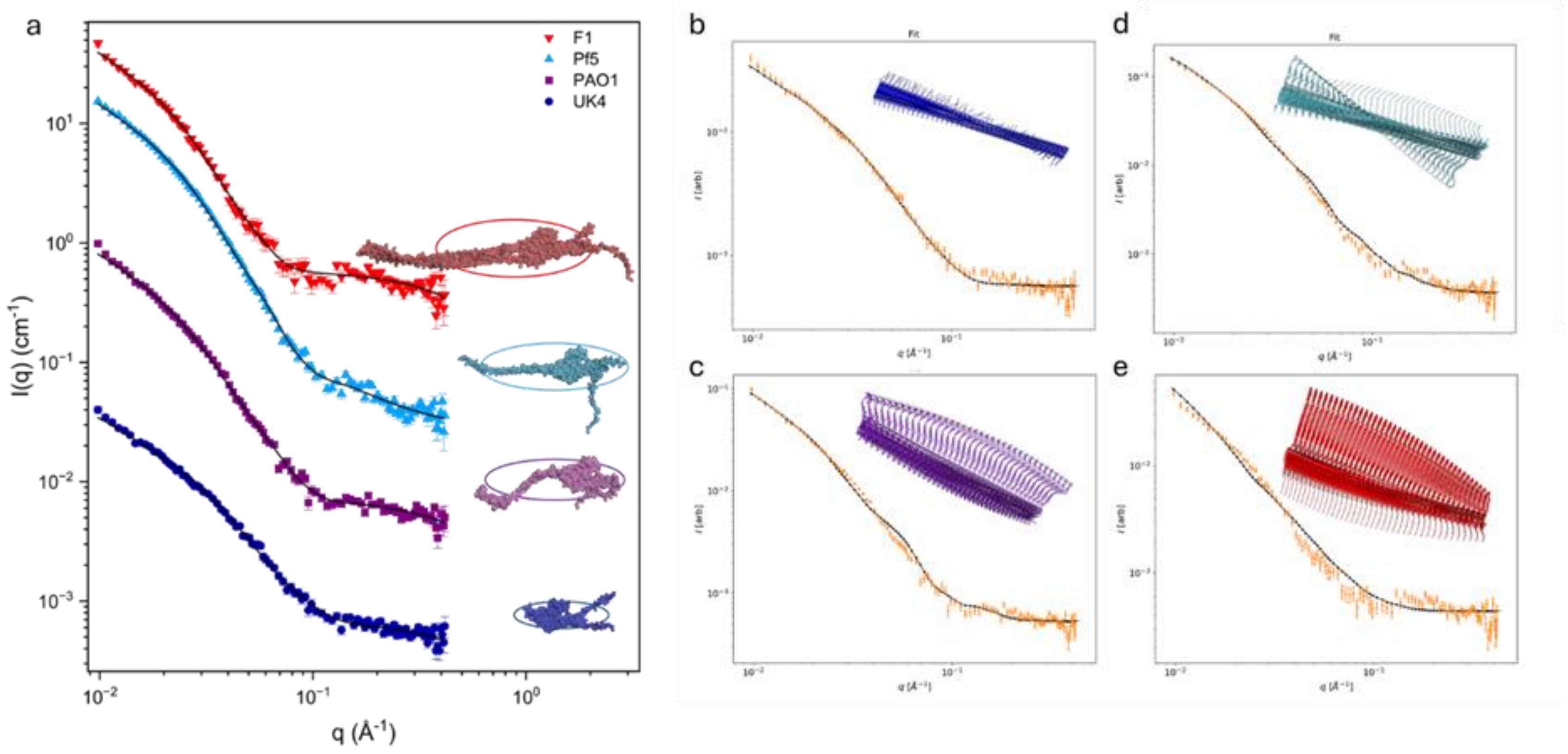

Figure S14. SAXS data and analysis results for FapC fibrils from the 4 different variants. a. Fit using the cylinder model. Included on the right side a depiction of the fibril cross section and the FA models for comparison. UK4 - Blue; PAO1 - Light blue; Pf5 - Purple; F1 - Red. The theoretical scattering calculated from each model fitting is shown as solid lines. Curves are stacked by a factor of 10 for clarity. **b-e.** All-atom modeling of single fibrils derived from the AF predictions and the corresponding fit. Each model is included as an inset with the same colur for clarity. Resulting reduced chi-squared χ^2 are: UK4 - 1.6, PAO1 - 6, PF5 - 9.3, F1 - 4.97.

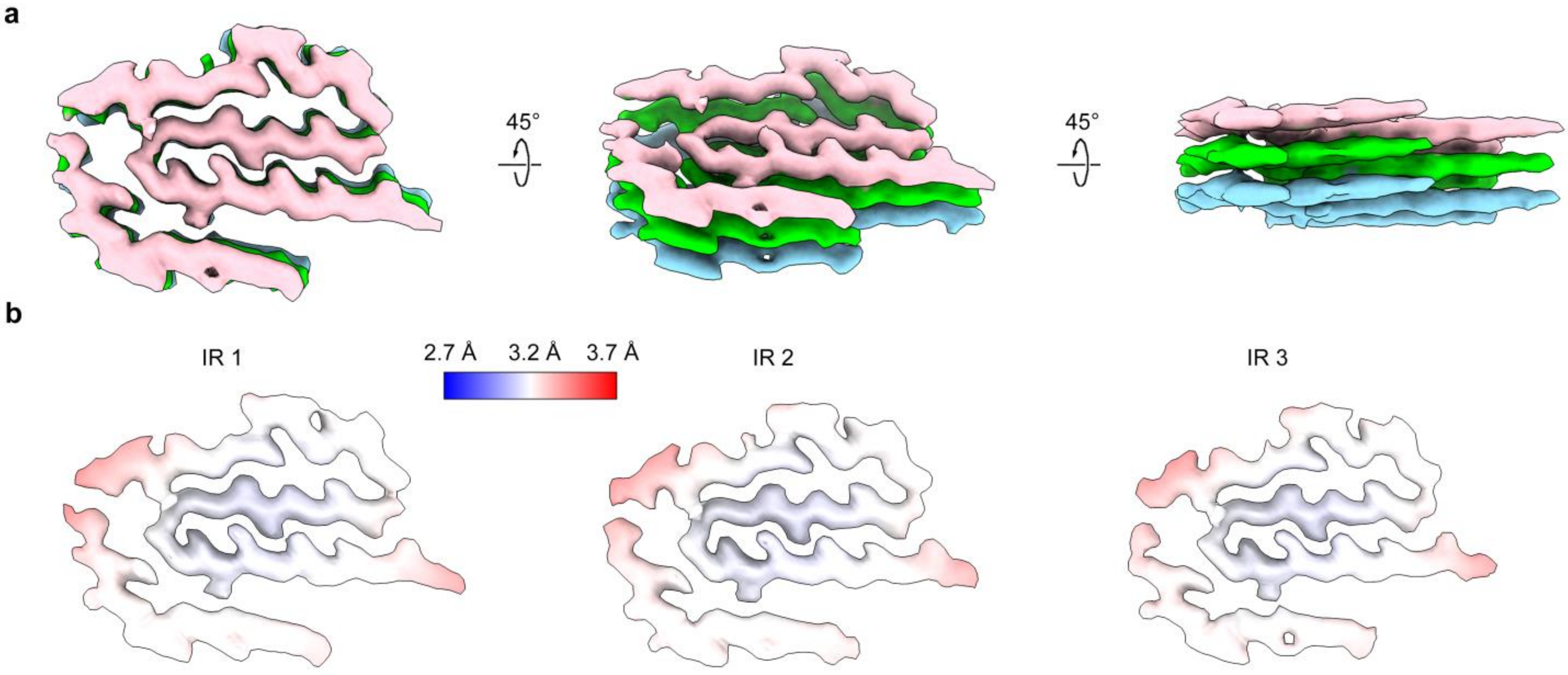

Figure S15. cryo-EM maps of FapC fibrils colored by imperfect repeats 1-3 (a) or local resolution (b).

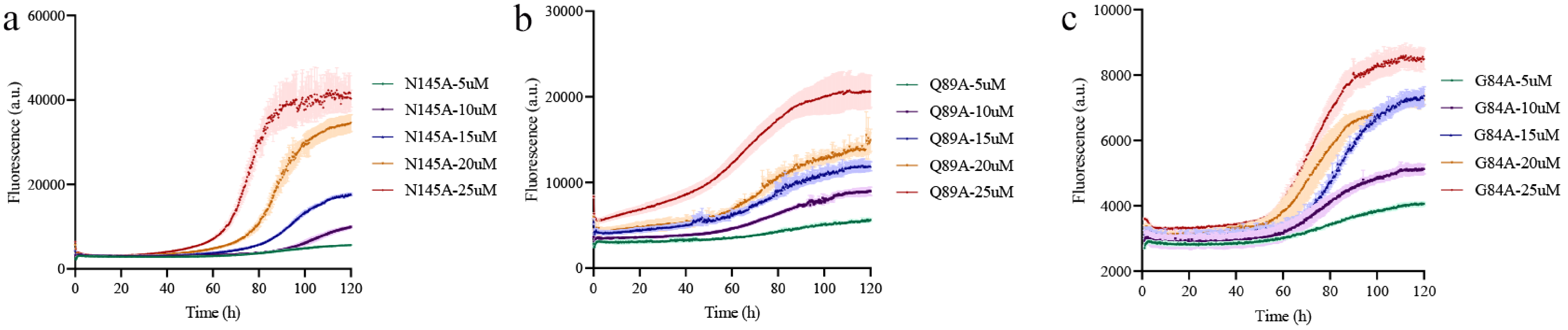

Figure S16. Kinetics time curve of N/Q/G mutant polymerization at different concentrations. The changes in ThT fluorescence intensity for (a) N145A, (b) Q89A, and (c) G84A at initial monomer concentrations ranging from 5 μM to 25 μM.

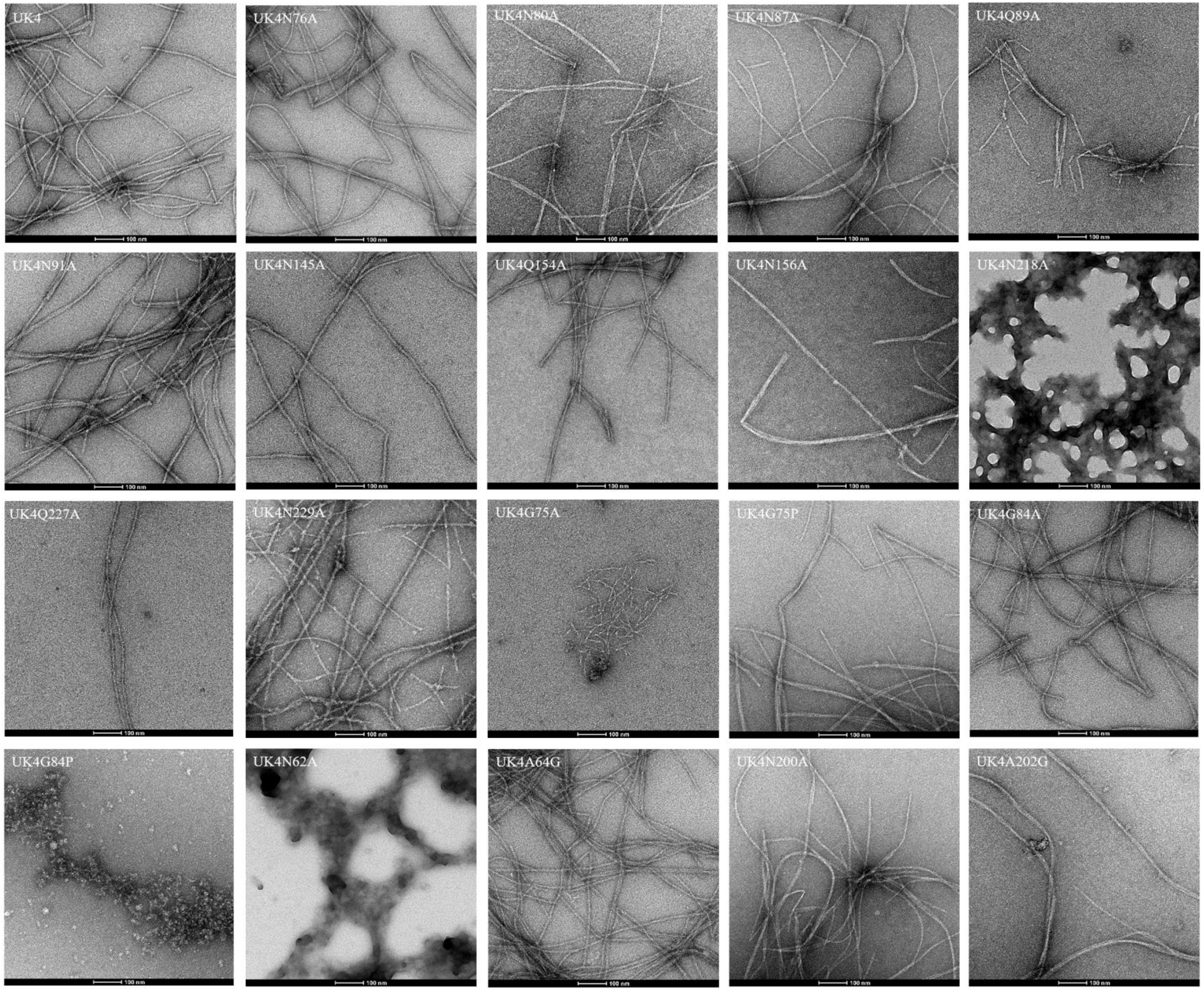

Figure S17. EM negative staining observation of fibril of UK4 N/Q/G mutants.

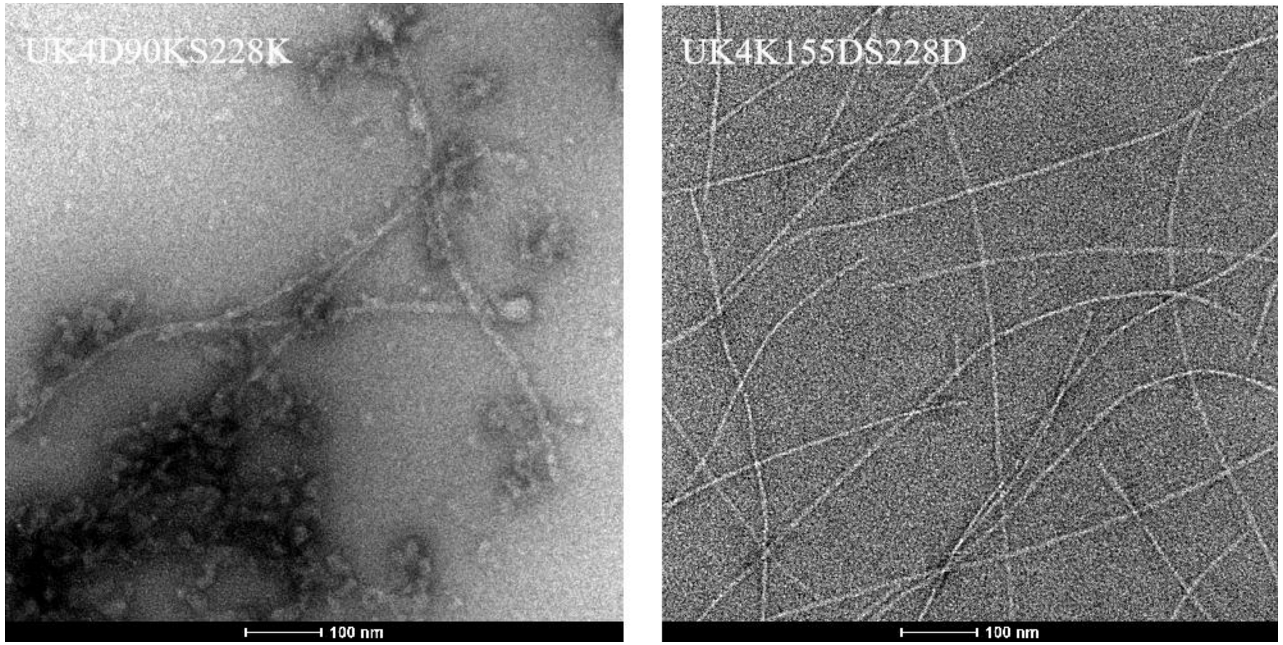

Figure S18. EM negative staining of fibrils of two different UK4 charge mutants.

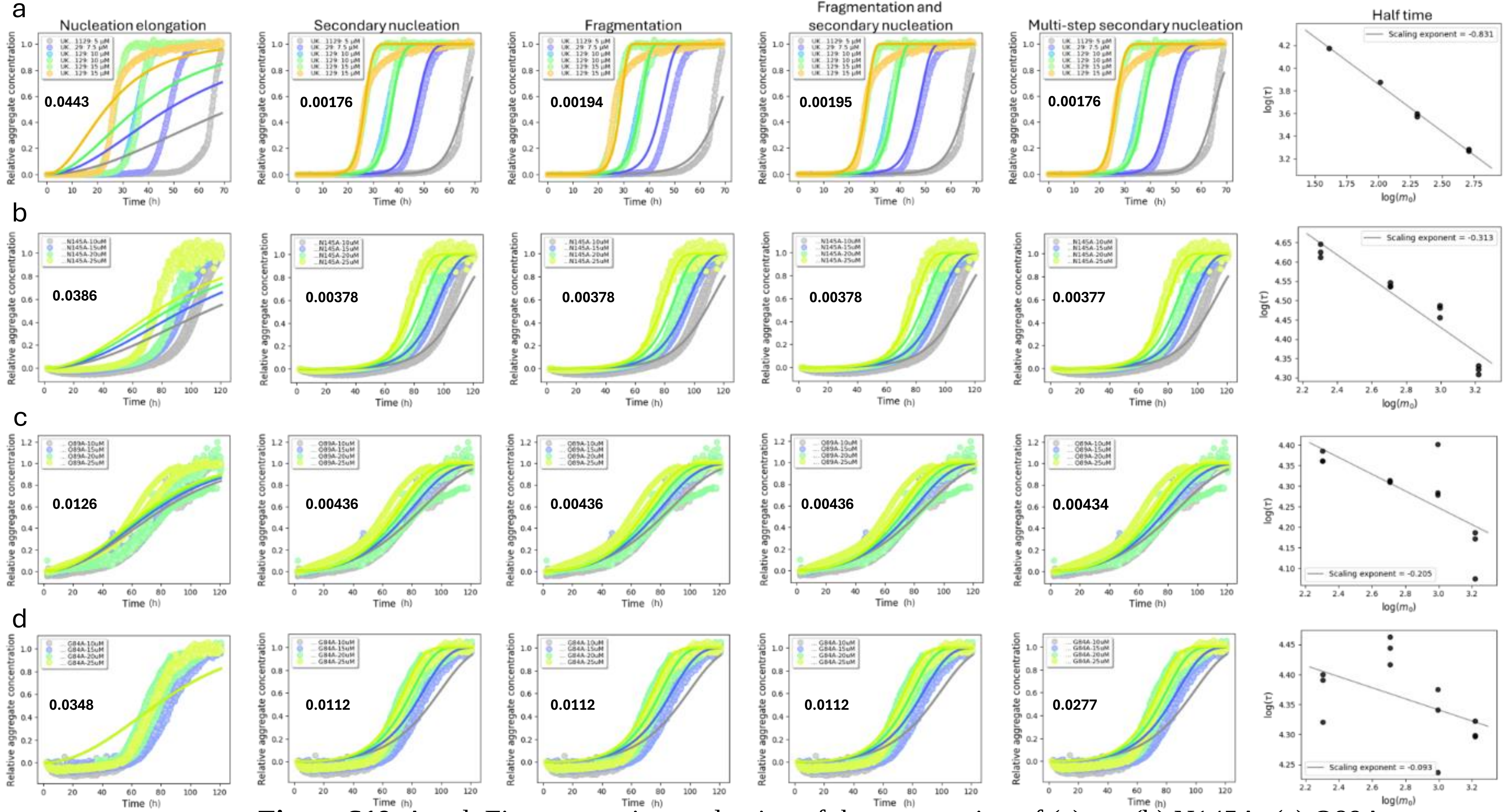

Figure S19. AmyloFit comparative evaluation of the aggregation of (a) wt, (b) N145A, (c) Q89A and (d) G84A FapC variants. Mean residue error (MRE) indicated for each fit in bold.

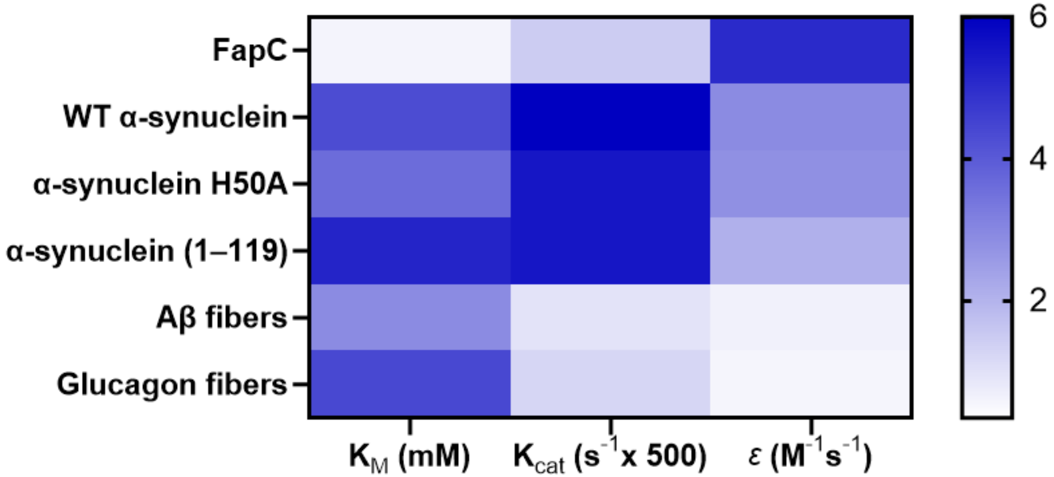

	Esterase			Lipase		
	K_M (mM)	K_{cat} (s^{-1})	ε ($M^{-1}s^{-1}$)	K_M (mM)	K_{cat} (s^{-1})	ε ($M^{-1}s^{-1}$)
**FapC**	0,5808	0,0029	5,0649	1,120	0,004	3,88
**WT α-synuclein**	4,3	0,012	2,9	-	-	-
**α-synuclein H50A**	3,6	0,011	2,8	-	-	-
**α-synuclein (1–119)**	5,2	0,011	2,1	-	-	-
**Aβ fibers**	2,9	0,0019	0,64	-	-	-
**Glucagon fibers**	4,4	0,0025	0,55	1,9	0,003	1,5

Figure S20. FapC catalytic constants compared to other amyloid fibrils. The data was derived from available literature (33, 37-39) and therefore not obtained from the same reaction conditions. Glucagon fibers lipase potential is evaluated in pNPP.

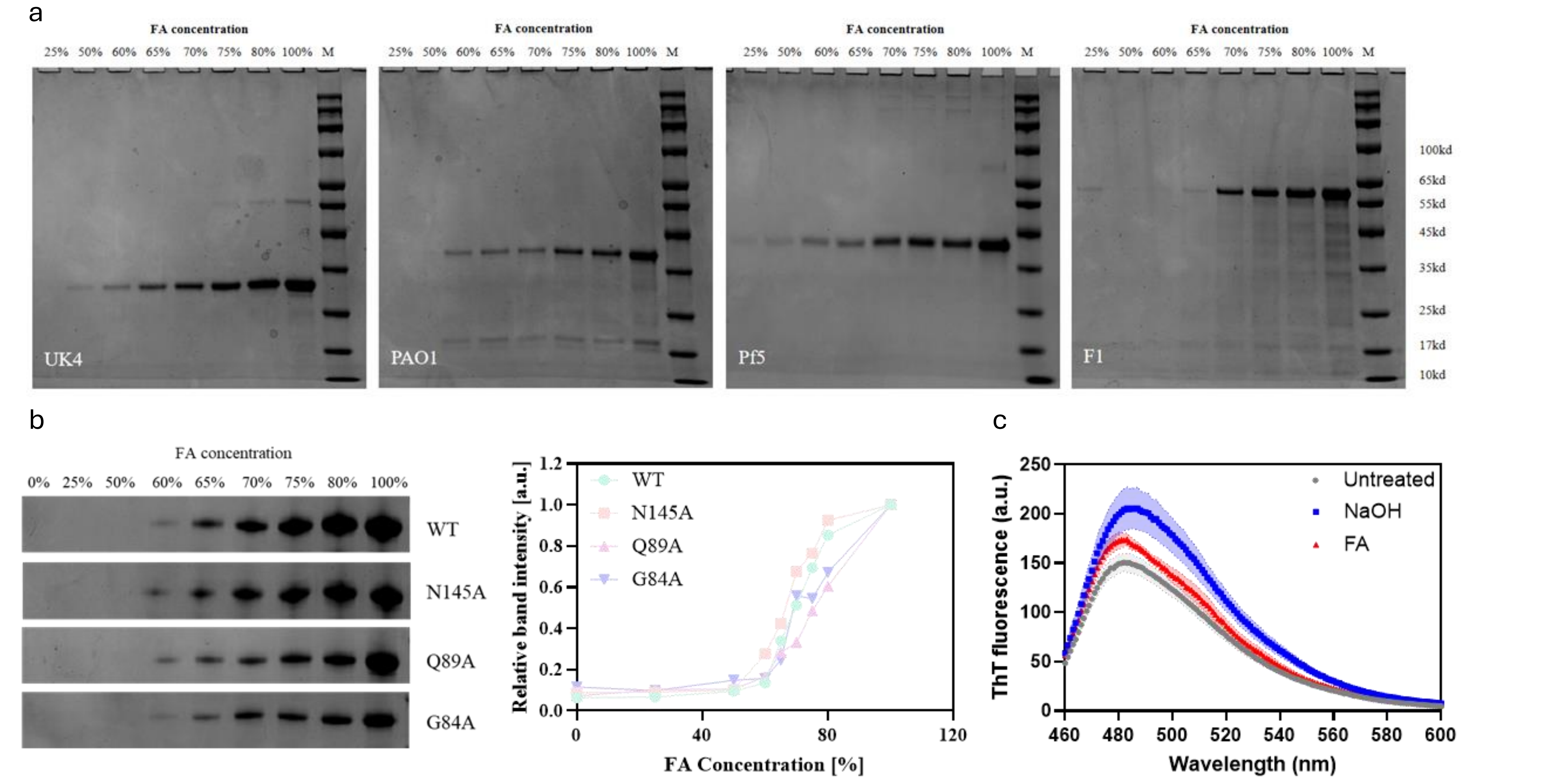

Figure S21. Formic acid stability of different FapC strains (a) and UK4 variant mutants (b). ThT derived fluorescence of FapC UK4 fibrils after incubation in alkaline and acidic conditions (c).

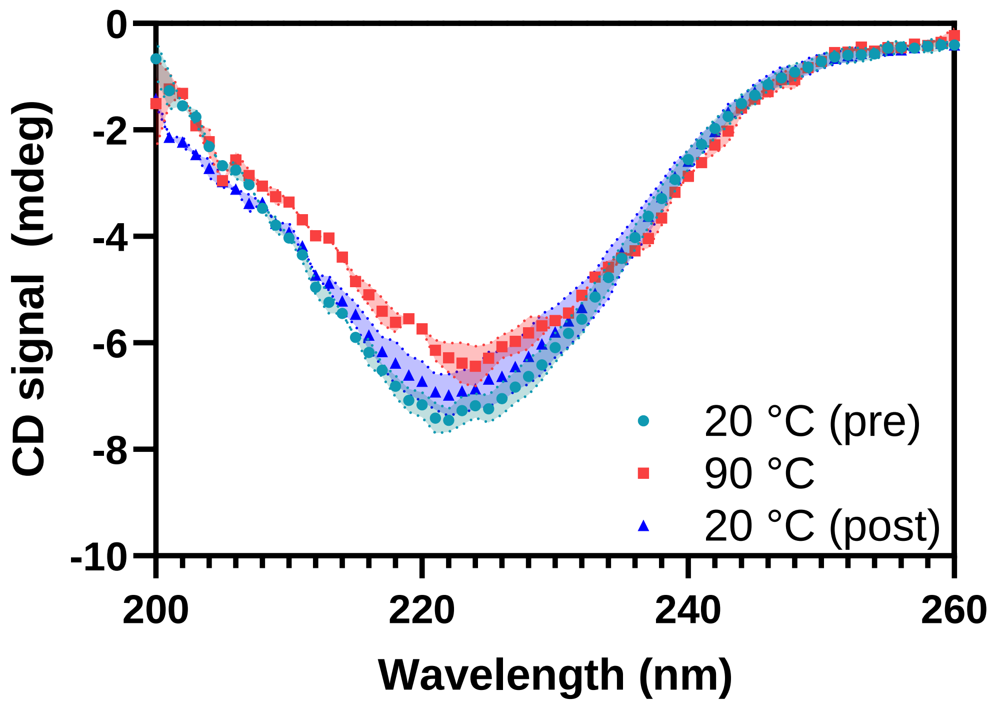

Figure S22. FapC fibrils do not change secondary structure upon heating. FapC UK4 fibrils were heated to 90°C at for 10 min, after which the spectrum was recorded at 90°C. After cooling down to 20°C and incubated for another 10 min, a final far-UV CD spectrum was recorded.

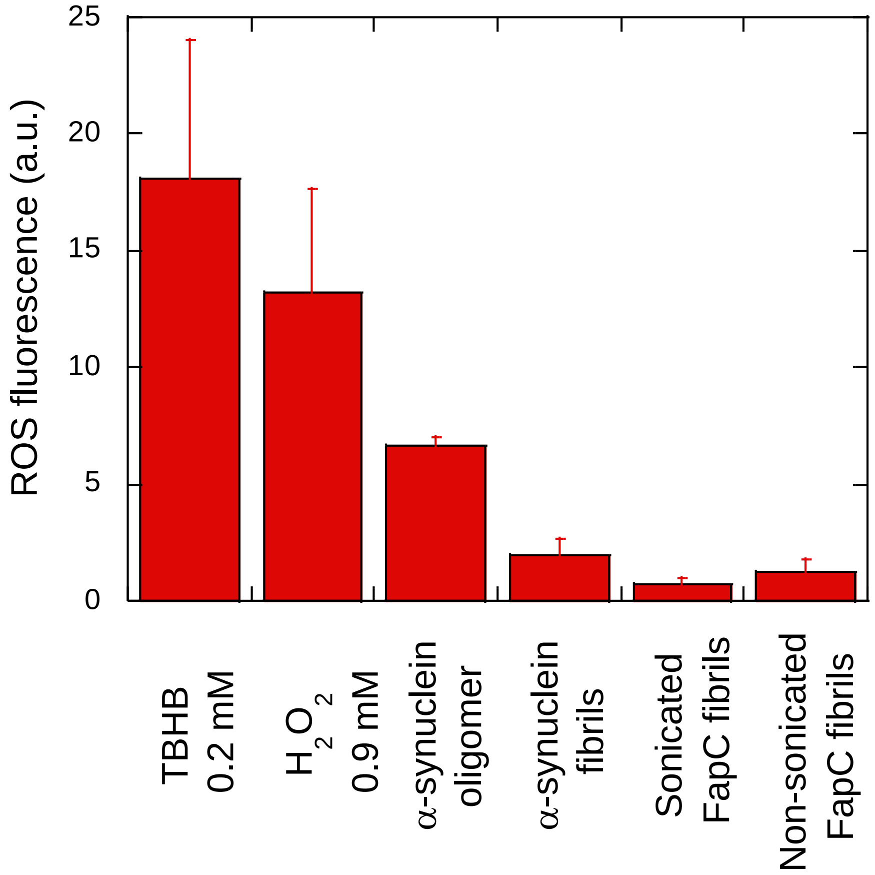

Figure S23. FapC fibrils are not harmful for human HEK293 cells. The data show the Reactive Oxygen Species (ROS) signal generated by HEK293 upon exposure to the oxidant tert-butyl hydroperoxide (THBP), the oxidant H_2O_2 and100 μg/ml each of α-synuclein oligomer and fibrils and fibrils of FapC. Control values (no fibrils or oxidants) are on level with FapC fibrils.

Table S1. AmyloFit-derived constants of UK4 seeded and cross-seeded reactions

	UK4				
	**Secondary + fragmentation**	**Fragmentation**	**PAO1**	**Pf5**	**F1**
k_n ($\mu M^{-nc-1}h^{-1}$)	3,1E-09	0,00000122	0,00689	0,358	0,00636
n_c (a.u.)	5,32	1,97	1,05	1	0,194
k_2 ($\mu M^{-n2}h^{-1}$)	0,0000237	-	-	-	-
n_2 (a.u.)	5,58E-10	-	-	-	-
k_+ ($\mu M^{-1}h^{-1}$)	3,56	1,97	0,000498	0,0000103	0,0000332
k_- (h^{-1})	0,00000191	0,000042	0,426	9,4	2,69
K_{off} (h^{-1})	3,41	0,00235	1,42E-10	3,28E-09	7,04E-12

Table S2. Values for the cross-section of FapC fibrils from SAXS data

	UK4		Pf5		F1		Pao1	
	Value	Error	Value	Error	Value	Error	Value	Error
R (nm)	1.67	0.13	2.9	0.08	3.52	0.15	2.49	0.09
ε	3.4	0.3	3.2	0.1	2.7	0.2	3.5	0.2
R_g (nm)	0.20	0.07	1.0	0.2	0.27	0.04	0.34	0.21
S_{pol}/S_{cyl}	0.070		0.0021		0.033		0.0064	

Table S3. Secondary structure contents of UK4 samples predicted by Dichroweb from Figure 12b

Species	Alpha helix	Beta sheet	Random coli
Monomer	0.16	0.44	0.40
Fibril	0.05	0.64	0.32

Table S4. Deconvolution of Fourier transform infrared (ATR-FTIR) spectroscopy from Figure 12c

Peak Index	Peak Type	Area Intg	FWHM	Max Height	Center Grvty	Area IntgP
1	Gaussian	5.9	20	0.27713	1628	67.0468
2	Gaussian	1	20	0.04697	1648	11.36386
3	Gaussian	1.3	20	0.06106	1661	14.77302
4	Gaussian	0.59982	20	0.02818	1681	6.81631
